# Comparative transcriptome analysis indicates that a core transcriptional network mediates isonuclear alloplasmic male sterility in wheat (*Triticum aestivum* L.)

**DOI:** 10.1186/s12870-019-2196-x

**Published:** 2020-01-07

**Authors:** Zihan Liu, Sha Li, Wei Li, Qi Liu, Lingli Zhang, Xiyue Song

**Affiliations:** College of Agronomy, Northwest A&F University, Yangling, Shaanxi China

**Keywords:** Isonuclear alloplasmic male sterility, Pollen abortion, Transcriptome-mediated network, Transcriptome sequencing, Wheat

## Abstract

**Background:**

Cytoplasmic male sterility (CMS) plays a crucial role in the utilization of heterosis and various types of CMS often have different abortion mechanisms. Therefore, it is important to understand the molecular mechanisms related to anther abortion in wheat, which remain unclear at present.

**Results:**

In this study, five isonuclear alloplasmic male sterile lines (IAMSLs) and their maintainer were investigated. Cytological analysis indicated that the abortion type was identical in IAMSLs, typical and stainable abortion, and the key abortive period was in the binucleate stage. Most of the 1,281 core shared differentially expressed genes identified by transcriptome sequencing compared with the maintainer in the vital abortive stage were involved in the metabolism of sugars, oxidative phosphorylation, phenylpropane biosynthesis, and phosphatidylinositol signaling, and they were downregulated in the IAMSLs. Key candidate genes encoding chalcone--flavonone isomerase, pectinesterase, and UDP-glucose pyrophosphorylase were screened and identified. Moreover, further verification elucidated that due to the impact of downregulated genes in these pathways, the male sterile anthers were deficient in sugar and energy, with excessive accumulations of ROS, blocked sporopollenin synthesis, and abnormal tapetum degradation.

**Conclusions:**

Through comparative transcriptome analysis, an intriguing core transcriptome-mediated male-sterility network was proposed and constructed for wheat and inferred that the downregulation of genes in important pathways may ultimately stunt the formation of the pollen outer wall in IAMSLs. These findings provide insights for predicting the functions of the candidate genes, and the comprehensive analysis of our results was helpful for studying the abortive interaction mechanism in CMS wheat.

## Background

Increasing crop yields in a sustainable and efficient manner is essential to meet the growing global demand for food [1]. Wheat (*Triticum aestivum* L*.*) is one of the most important grain and forage crops throughout the world, where it accounts for 20% of the total energy consumed by humans. However, wheat breeding is still underdeveloped and only a small amount of the available diversity is exploited at present [2]. Hybrid wheat breeding is a highly promising strategy for maximizing the use of heterosis to increase wheat yields [3]. In particular, the cytoplasmic male sterility (CMS) system is based on incompatible interactions between the nucleus and cytoplasm, and it is commonly used in many species for hybrid seed production. Moreover, CMS comprises the maternally inherited inability to produce functional pollen in some crop plants, thereby providing an ideal material for studying cytoplasmic inheritance, reproductive growth, and pollen development [4]. CMS is a very powerful tool for hybrid wheat breeding, but the mechanism responsible for pollen abortion in CMS remains unclear at present. Furthermore, CMS lines with different types of germplasm often possess diverse abortion mechanisms, so it is particularly important to determine their core and common abortion mechanism. In previous studies, we showed that an ideal set of isonuclear alloplasmic male sterile lines (IAMSLs), i.e., K706A with *Aegilops kotschyi* cytoplasm, Va706A with *Ae. vavilovii* cytoplasm, Ju706A with *Ae. juvenalis* cytoplasm, C706A with *Ae. crassa* cytoplasm, and U706A with *Ae. uniaristata* cytoplasm, have many superior advantages that can be utilized in hybrid wheat production, where they can improve the wheat quality, are easy to restore, enhance powdery mildew resistance, and increase the growth potential [5]. Thus, the cytological and physiological mechanisms related to pollen abortion have been investigated in these IAMSLs [6]. However, the molecular mechanisms related to CMS in these five types of IAMSLs are still unclear, especially their core molecular mechanisms. Therefore, elucidating the common molecular mechanism responsible for pollen abortion in these IAMSLs will provide a theoretical basis to facilitate hybrid wheat breeding.

In recent years, transcriptome sequencing has been employed as a powerful tool for studying global transcription networks to provide high resolution data and it has been applied widely in many crops, such as soybean [7], cotton [8], and rice [9]. In addition, it has been employed to investigate leaf senescence [10], leaf color [11], biotic stress responses [12], and abiotic stress responses [13] in wheat. However, few transcriptome analyses have considered pollen abortion and male sterility in wheat. In general, the CMS plants could produce non-functional anther and pollen because of the interactions between mitochondrial and nuclear genes [14]. More specifically, in plants, the anther and pollen developmental processes are highly complex, where they involve the expression, regulation, metabolism, and activity of many genes. If these processes become disordered, then pollen development are blocked, thereby causing pollen abortion and CMS. In eggplants, 1,716 genes related to pollen development are mainly involved in male sterility, including genes related to oxidation-reduction, carbohydrate and amino acid metabolism, and transcriptional regulation [15]. In cabbage (*Brassica campestris* L*.*), many key genes are also involved in pollen and anther development, such as *MYB39*, *A6*, *AMS*, *MS1*, and *TSM1*, which are associated with tapetum development and pollen wall formation and the changes of these genes may be important cause of pollen abortion and male sterility [16]. In plants, the development of pollen and pollen wall requires the accumulation of starch, which is used as an energy reserve in the later stages of germination. However, imbalances in sugar metabolism and synthesis can severely damage pollen development, thereby leading to male sterility [17]. In rice and *Arabidopsis*, UDP-glucose pyrophosphorylase1 (UGP1) and UDP-glucose pyrophosphorylase2 (UGP2) involving in sugar metabolic pathway are essential for pollen starch accumulation and callose deposition, and their suppression results in pollen abortion and male sterility [18, 19]. Mitochondria are important sites for respiratory electron transfer, the generation of reactive oxygen species (ROS), and ATP synthesis [20]. In eukaryons, oxidative phosphorylation is thought to be the main pathway responsible for ATP production in respiratory chain electron transport. Moreover, the rearrangement and recombination of mitochondrial genomes often may lead to excess ROS accumulation and inhibition of ATP synthesis, thereby causing CMS [21]. In pepper, the open reading frames (*orf*) of mitochondrial F0 ATP synthase complex gene, such as *atp6*, have been identified as one of the candidates for CMS pepper by RNA editing and northern blot analysis, and further indicating the CMS trait is related to mitochondrial dysfunction [22]. Previous research also showed superabundant ROS would result in lipid peroxidation and oxidative damage of cells, protein and DNA damage, and even causing abnormal tapetal programmed cell death (PCD) [23, 24]. During the pollen development process, the phenylpropanoid biosynthesis pathway plays a critical role, and the key phenylpropanoid enzymes such as phenylalanine ammonia-lyase and cinnamyl alcohol dehydrogenase participate in sporopollenin formation, which is secreted into the anther chamber. And appropriate tapetal PCD can provide sufficient sporopollenin to maintain normal pollen wall development [25]. Therefore, the normal expression and regulation of the genes containing sugar metabolic pathway, oxidative phosphorylation pathway, and phenylpropanoid biosynthesis are the basis for the formation and development of pollen. In contrast, once these pathways are disturbed, CMS may be triggered. It is worth noting several genes with important roles in CMS have been identified but how these genes mediate metabolic-induced male sterility still needs to be elucidated, and the regulatory network related to CMS is largely unknown, especially in wheat.

In the present study, we used five types of IAMSLs and their maintainer line to obtained a comprehensive overview of the cellular and metabolic changes related to pollen abortion, and a core transcriptional regulatory network in male sterile wheat was further constructed by bioinformatics analyses and various experimental verification. Meanwhile, these findings provide novel insights into the core mechanism of CMS in wheat and the pollen development process.

## Results

### Identification of morphological defects and abortive stage in the IAMSLs

**Fig. 1:**
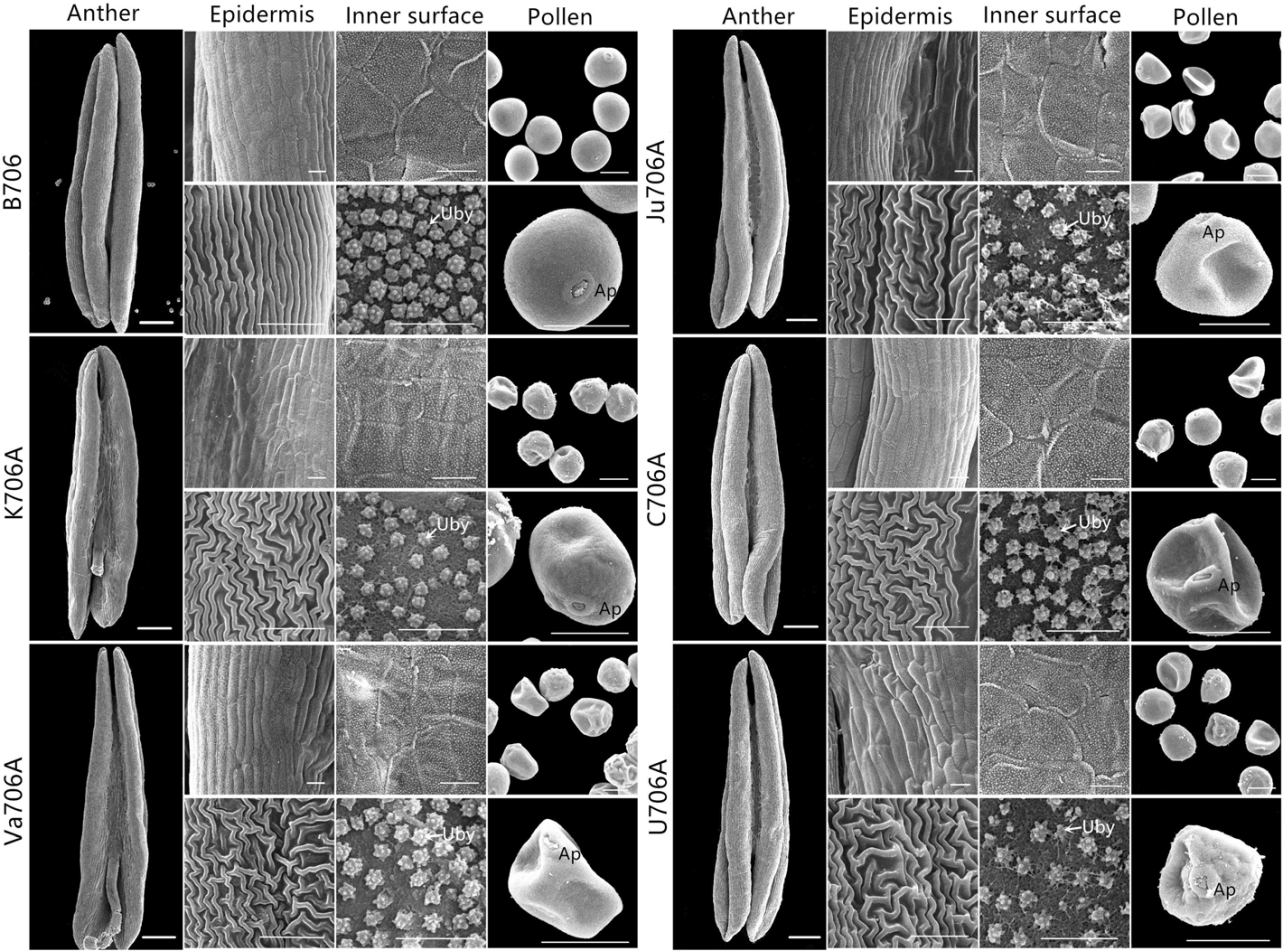
Comparison of scanning electron micrograph images of the anther, epidermis, inner surface, and pollen in the IAMSLs and B706 at the trinucleate stage. Uby: Ubisch bodies; Ap; germination aperture. Scale bars represent 1 mm in anthers, 50 μm in pollen, and 10 μm on the epidermis and inner surface

**Fig. 2:**
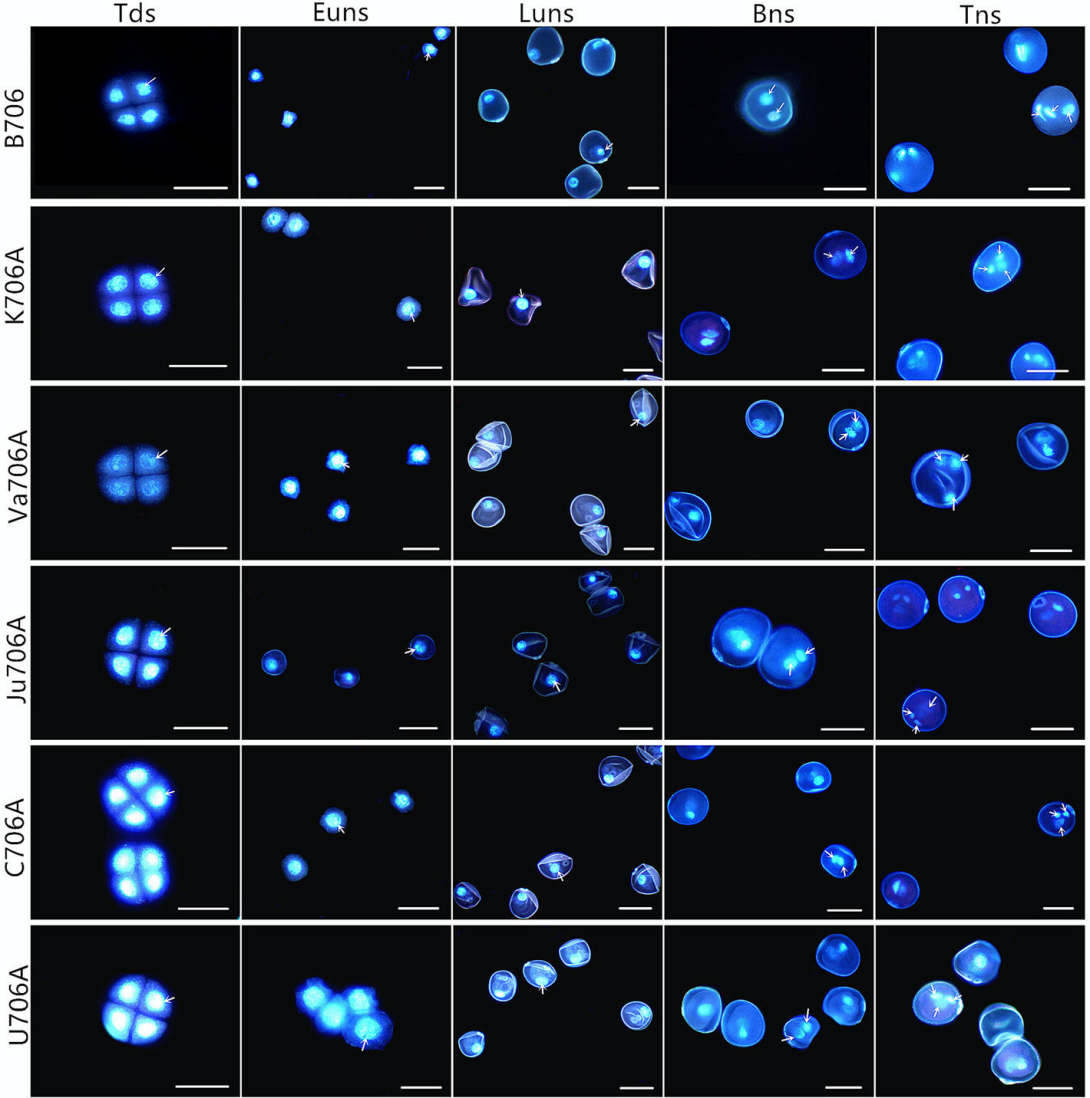
DAPI staining of microspores in the IAMSLs and B706 during various developmental stages. Tds, tetrad stage; Euns, early uninucleate stage; Luns, late uninucleate stage; Bns, binucleate stage; Tns, trinucleate stage. Scale bars represent 50 μm. The white asterisk denotes sperm-nucleus

The phenotypic characteristics of anthers from the IAMSLs (i.e., K706A, Va706A, Ju706A, C706A, and U706A) and B706 in various developmental stages were observed by stereomicroscopy and scanning electron microscopy. Compared with the IAMSLs, at the trinucleate stage, the anthers from B706 were normally dehiscent with the shedding of mature pollen grains, whereas the anthers did not crack in the IAMSLs. Unlike the mature fertile plants, the epidermis cell and inner epidermal Ubisch bodies in the IAMSLs had irregular shapes with a disordered arrangement (Fig. 1 and Additional file 1: Figure S1). Ubisch bodies are formed by the fusion of precursor vesicles containing sporopollenin and plasma membrane of tapetum cells and then secreting on the outside tapetum cells. The main function of Ubisch bodies is to transport the substances involved in the formation of pollen walls between tapetum and pollen, so the abnormal Ubisch bodies may affect the development of pollen. Furthermore, we used I_2_– KI staining to observe the pollen grains of the trinucleate stage, in contrast to B706, the pollen was 100% sterile in all of the IAMSLs and the pollen abortion type was typical and stainable abortion (Additional file 1: Figure S1). In addition, the microspores and growth of the nuclei were observed to further clarify the abortive critical period for the sterile plants based on 4′,6-diamidino-2-phenylindole (DAPI) staining. There were no clear differences in the tetrad stage and early uninucleate stage among all of the materials. However, the microspores from the IAMSLs were highly corrugated and they began to exhibit the characteristic features of sterility in the late uninucleate stage. Subsequently, compared with B706, in the binucleate stage, the vegetative nuclei were unclear and the microspore showed obvious abortion characteristics in the IAMSLs. Up to the trinucleate stage, the spindle sperm nuclei were substituted by nuclei with an abnormal circular shape (Fig. 2). Therefore, we found that the vital period for male abortion was identical in the IAMSLs, where it appeared to occur at binucleate stage according to the markedly aberrant morphology of the pollen and features of the nuclei. The results also indicated that the five types of IAMSLs were completely sterile until the trinucleate stage.

### Transcriptome sequencing

Three individual biological replicates of anthers in the binucleate stage from the five types of IAMSLs and B706 were used for deep RNA-seq analysis. After a stringent quality filtering process, 122.59 Gb of high-quality clean data were obtained. The percentage of reads with an average quality score > 30 from each sample was ≥78.46%, and the GC% for the clean data ranged from 55.12 to 59.82%. On average, 77.68% of the clean reads were mapped to the wheat reference genome and the known genes (83.98–86.09% of the total model genes) were generated by assembling these clean reads (Additional file 3: Table S2). By statistical analysis of the gene expression distribution of each sample, the results showed that the gene percentage distribution of different expression levels in a single sample were consistent (Additional file 1: Figure S2). FPKM violin analysis based on all of the samples detected no bias in the construction of the cDNA libraries and the gene expression levels in each sample (Additional file 1: Figure S3). Correlation heat map analysis detected high correlations between the biological replicates, thereby indicating that the sample selection process was reasonable and the sequencing data were reliable (Additional file 1: Figure S4). Therefore, the quality and accuracy of the sequencing data were adequate for further analysis.

### Analysis of differentially expressed genes

**Fig. 3:**
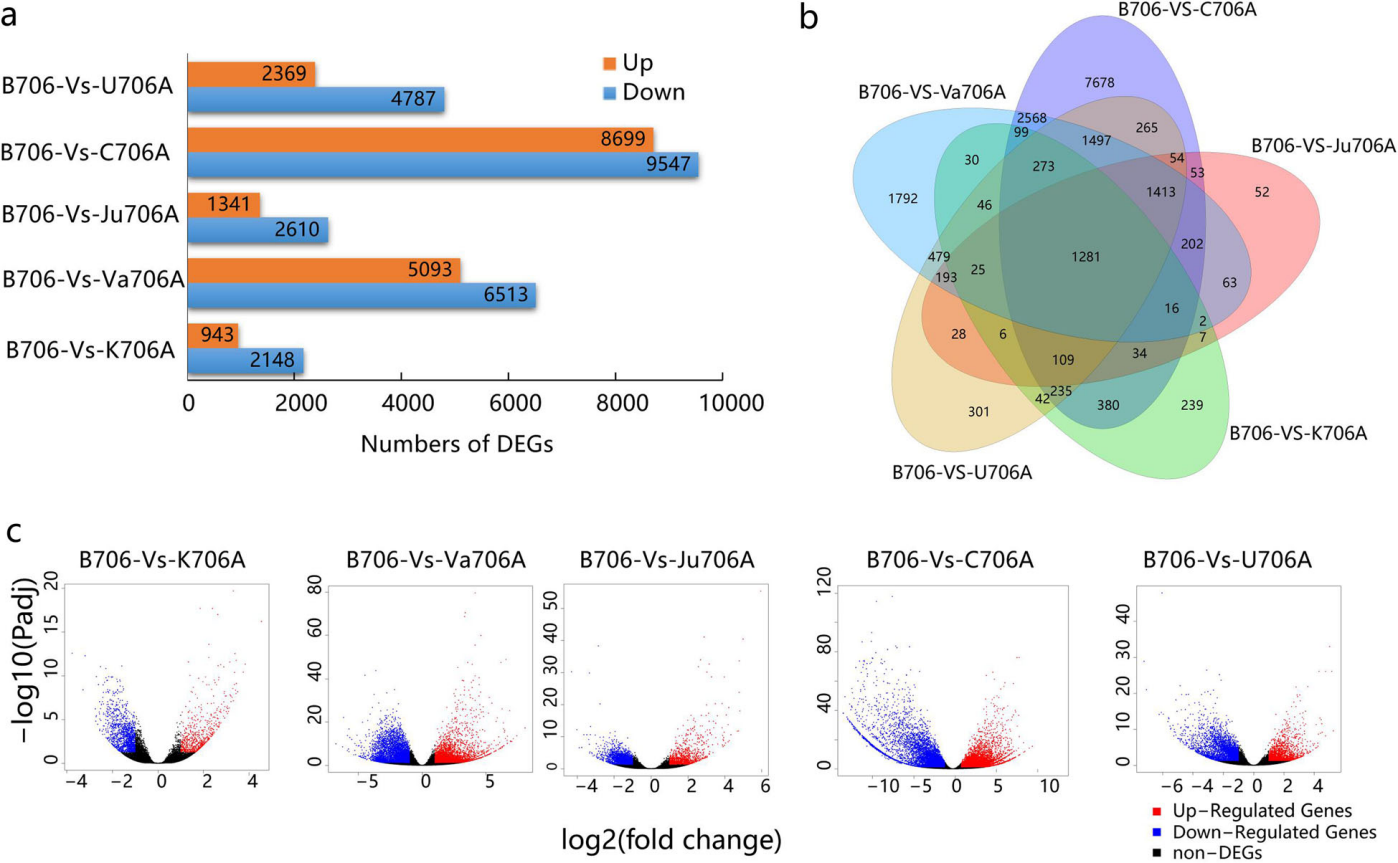
Analysis of DEGs in the five types of IAMSLs and B706. **a** Histogram showing the number of DEGs in the IAMSLs. **b** Venn diagram showing the mutual overlaps of DEGs in the IAMSLs. **c** Volcano plots showing the expression levels of every gene in the core set of shared DEGs

Differentially expressed genes (DEGs) were screened based on set criteria (FDR < 0.05 and |Log2FC| > 1) in each sterile line and the maintainer line (B706 vs. K706A, B706 vs. Va706A, B706 vs. Ju706A, B706 vs. C706A, and B706 vs. U706A). Compared with B706, 3,091 DEGs were identified in K706A, as well as 11,606 DEGs in Va706A, 3,951 DEGs in Ju706A, 18,246 DEGs in C706A, and 7,156 DEGs in U706A (Fig. 3a). In order to explore the common molecular mechanisms related to CMS abortion, we compared the DEGs by investigating the shared genes in the five types of IAMSLs. The Venn diagram in Fig. 3b shows that there was a large amount of overlap between the DEGs in the IAMSLs, where a core set comprising 1281 DEGs had similarly different expression levels in all five types of IAMSLs (Additional file 4: Table S3). Interestingly, this core set of shared DEGs comprised 158 upregulated DEGs and 1,123 downregulated DEGs in each sterile material, but the actual gene expression levels and significant differences in the DEGs were different (Fig. 3c). We also found that most of the shared DEGs were downregulated in the IAMSLs, including chalcone synthase (*TraesCS5A02G379400*, *TraesCS5B02G383300*), ABC transporter (*TraesCS7B02G488900, TraesCSU02G106900*), and cytochrome P450 (*TraesCS4A02G358200*, *TraesCS5B02G514200, TraesCS3A02G505000*) (Additional file 4: Table S3). Previous studies have shown that the inhibition of these genes affects pollen exine formation, thereby leading to male sterility [26]. Thus, it was necessary to analyze the shared DEGs in the five IAMSLs in order to explore the common causes of abortion in CMS wheat because the downregulation of most of the core set of genes may be related to male sterility in the IAMSLs.

### Functional classification and enrichment analysis of core DEGs

To further clarify the functions of the core DEGs, KOG classifications were obtained for the annotated gene sequences. In total, 509 DEGs were annotated according to 23 KOG categories, where “General function prediction only” with 212 ranked highest. Moreover, “Signal transduction mechanisms” was represented by 144 DEGs, followed by “Transcription” with 99 DEGs (Additional file 1: Figure S5). Therefore, we inferred that a large number of changes in signal transduction-related genes possibly hindered signal transfer in the cell to eventually lead to sterility in the IAMSLs.

**Fig. 4:**
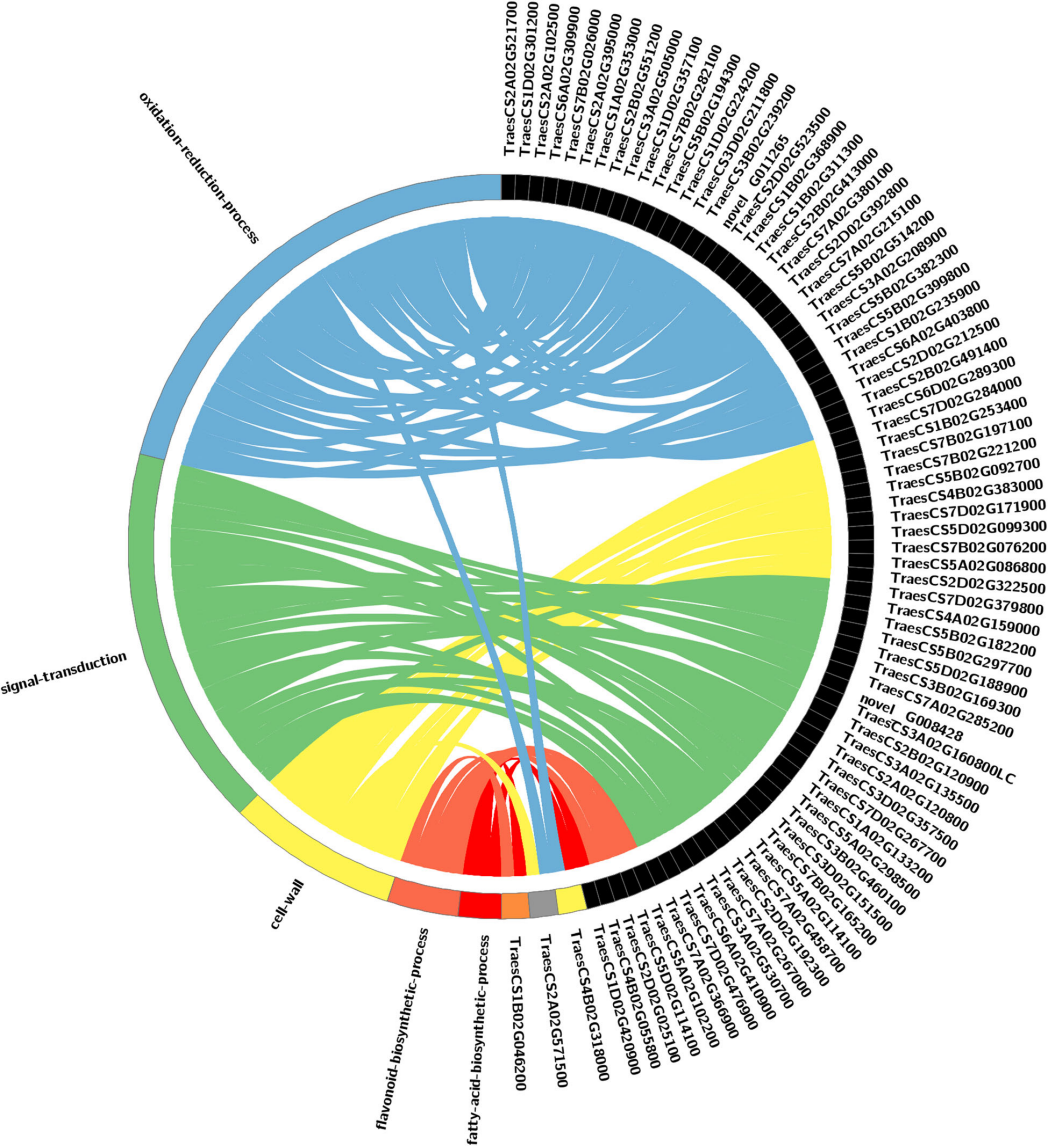
Association graph showing the relationships between important GO terms and the related core DEGs. Important GO terms include: GO: 007165, signal transduction; GO: 0055114, oxidation-reduction process; GO: 0009813, flavonoid biosynthetic process; GO: 0006633, fatty acid biosynthetic process; and GO: 0005618, cell wall

The core DEGs annotated using the GO database were classified into three principal categories from three ontologies: “biological process”, “cellular component”, and “molecular function”. According to the GO classifications at four levels, enrichment of the core DEGs demonstrated that 44 GO terms were significant (*p* < 0.01) (Additional file 1: Figure S6). Furthermore, the tertiary and quaternary functional classifications demonstrated that five GO terms comprising “signal transduction”, “oxidation-reduction process”, “flavonoid biosynthetic process”, “fatty acid biosynthetic process,” and “cell wall” were dominant in the DEGs shared by the five CMS lines (Additional file 1: Figure S7A–C). The relationships between the five important GO terms and the related DEGs were also analyzed based on an association graph. As shown in Fig. 4, a gene annotated as pectinesterase (*TraesCS2A02G571500*) was associated with both “cell wall” and “oxidation-reduction process”. Indeed, a previous study demonstrated that pectinesterase is associated with pollen fertility, pollen germination, and anther dehiscence in *Arabidopsis thaliana* [27]. According to the present study, the 13 DEGs annotated as pectinesterase may control pollen cell wall formation during pollen abortion in the IAMSLs. In addition, a gene (*TraesCS1B02G046200*) annotated as the chalcone--flavonone isomerase was included in two GO terms (“flavonoid biosynthetic process”, and “fatty acid biosynthetic process”), thereby indicating that this gene probably plays a vital role in CMS wheat. These results indicate that pollen abortion in CMS wheat might be related to changes in genes associated with the cell wall, thereby affecting pollen wall formation and anther dehiscence to ultimately lead to sterility.

**Fig. 5:**
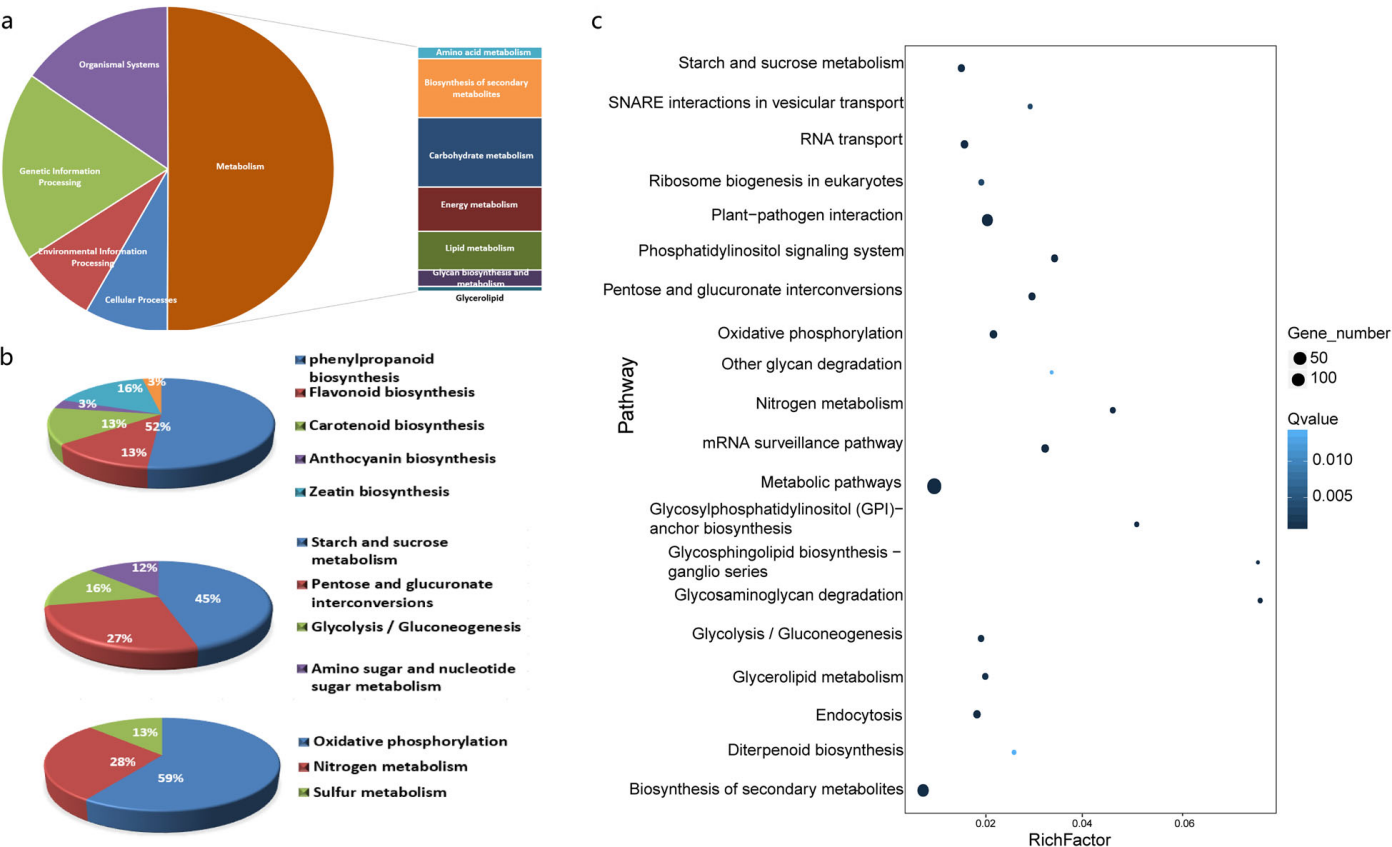
KEGG enrichment of core DEGs in all five types of IAMSLs. **a** Primary and secondary classification based on KEGG. **b** Classification based on biosynthesis of secondary metabolites, energy metabolism and carbohydrate metabolism. **c** Bubble diagram illustrating the KEGG enrichment results. Rich factor is the ratio of DEG numbers annotated for a pathway term relative to all genes annotated for this pathway term. A highest Rich factor indicates a greater degree of pathway enrichment. A lower *p*-value indicates greater pathway enrichment

**Fig. 6:**
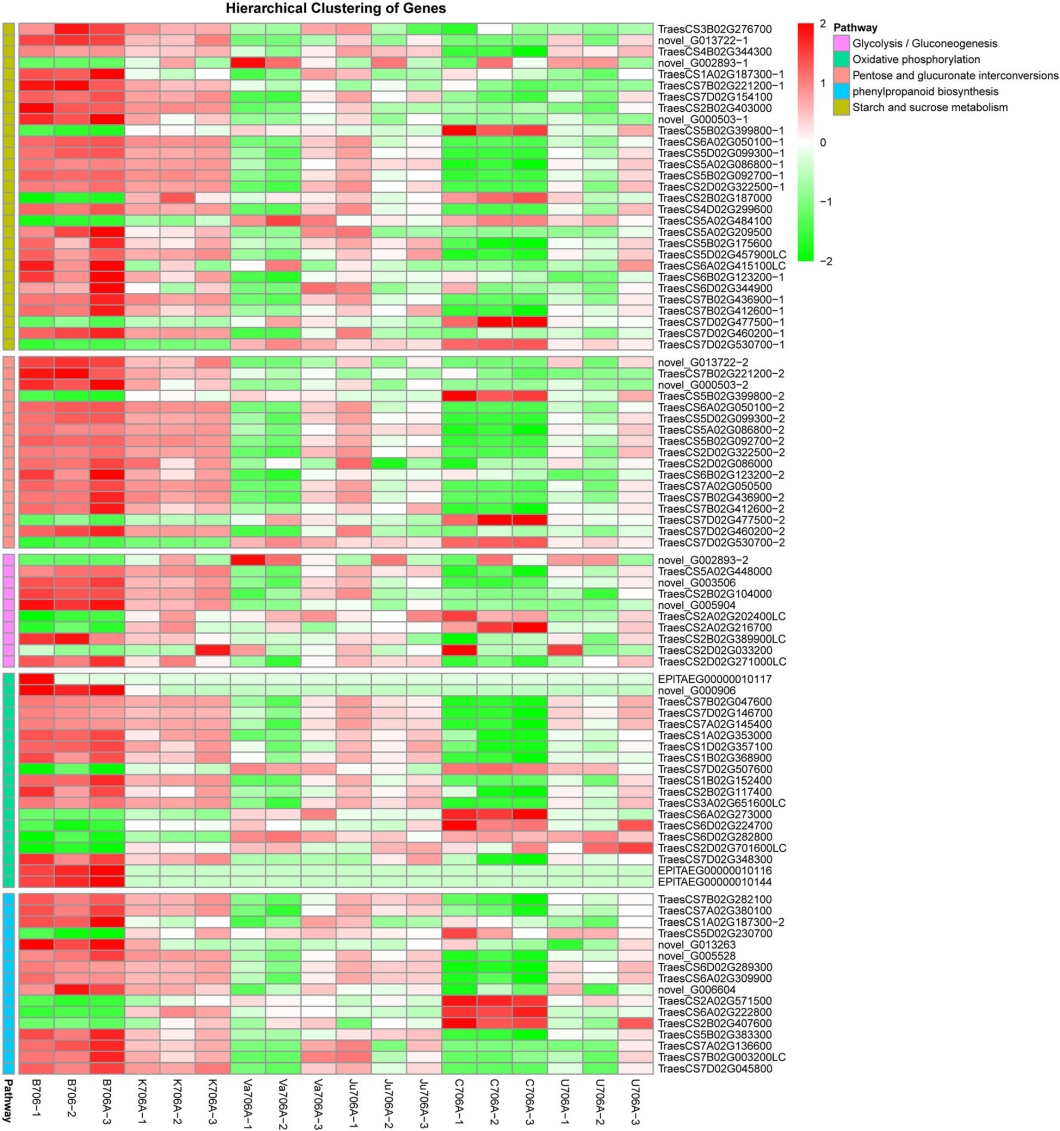
Hierarchical clustering analysis of core DEGs from the biosynthesis of secondary metabolites, carbohydrate metabolism and energy metabolism pathways

KEGG enrichment analysis was performed to further identify the functions of the core DEGs in specific biological pathways. The annotated DEGs were assigned to 81 KEGG pathways, where “pentose and glucuronate interconversions”, “starch and sucrose metabolism”, and “glycolysis/gluconeogenesis” were the extremely representative based on the bubble diagram. Interestingly, similar to the KOG and GO results where the “signal transduction” category occupied the main position, the “phosphatidylinositol (PI) signaling system” pathway was also significant according to KEGG enrichment analysis (Fig. 5a-c). Many previous studies have also suggested that genes and enzymes related to phosphatidylinositol have regulatory effects on male sterility [28, 29]. The KEGG classifications at three levels also showed that pathways related to “biosynthesis of other secondary metabolites”, “carbohydrate metabolism” and “energy metabolism” were associated with many of the DEGs, such as “phenylpropanoid biosynthesis”, “starch and sucrose metabolism”, “glycolysis/gluconeogenesis”, and “oxidative phosphorylation” (Fig. 5a-c). Hierarchical clustering analysis was applied to the DEGs in the major pathways (Fig. 6). Compared with B706, most of the DEGs in these major pathways were downregulated in the IAMSLs. Moreover, as shown in Fig. 6, some DEGs were strongly downregulated, such as the genes encoding UTP--glucose-1-phosphate uridylyltransferase (*TraesCS6A02G050100*) and putative pectinesterase 53 (*TraesCS5A02G086800*, *TraesCS5B02G092700, TraesCS5D02G099300*) in “pentose and glucuronate interconversions” and “starch and sucrose metabolism”, pyruvate decarboxylase (*TraesCS2B02G104000*) in “glycolysis/gluconeogenesis” and (*TraesCS2D02G086000*) in “pentose and glucuronate interconversions”, peroxidase (*TraesCS6A02G309900, TraesCS6D02G289300*) in “phenylpropanoid biosynthesis”, and atp6–1 (*EPlTAEG00000010144* and *EPlTAEG00000010116*) in “oxidative phosphorylation”, which are associated with metabolic regulation, pollen development and energy synthesis. These genes were severely suppressed in the IAMSLs, which indicates that they may be related to male sterility, thereby providing a valuable resource for studying male sterility in CMS wheat.

### Identification of male sterility-related hub DEGs by weighted gene co-expression network analysis

**Fig. 7:**
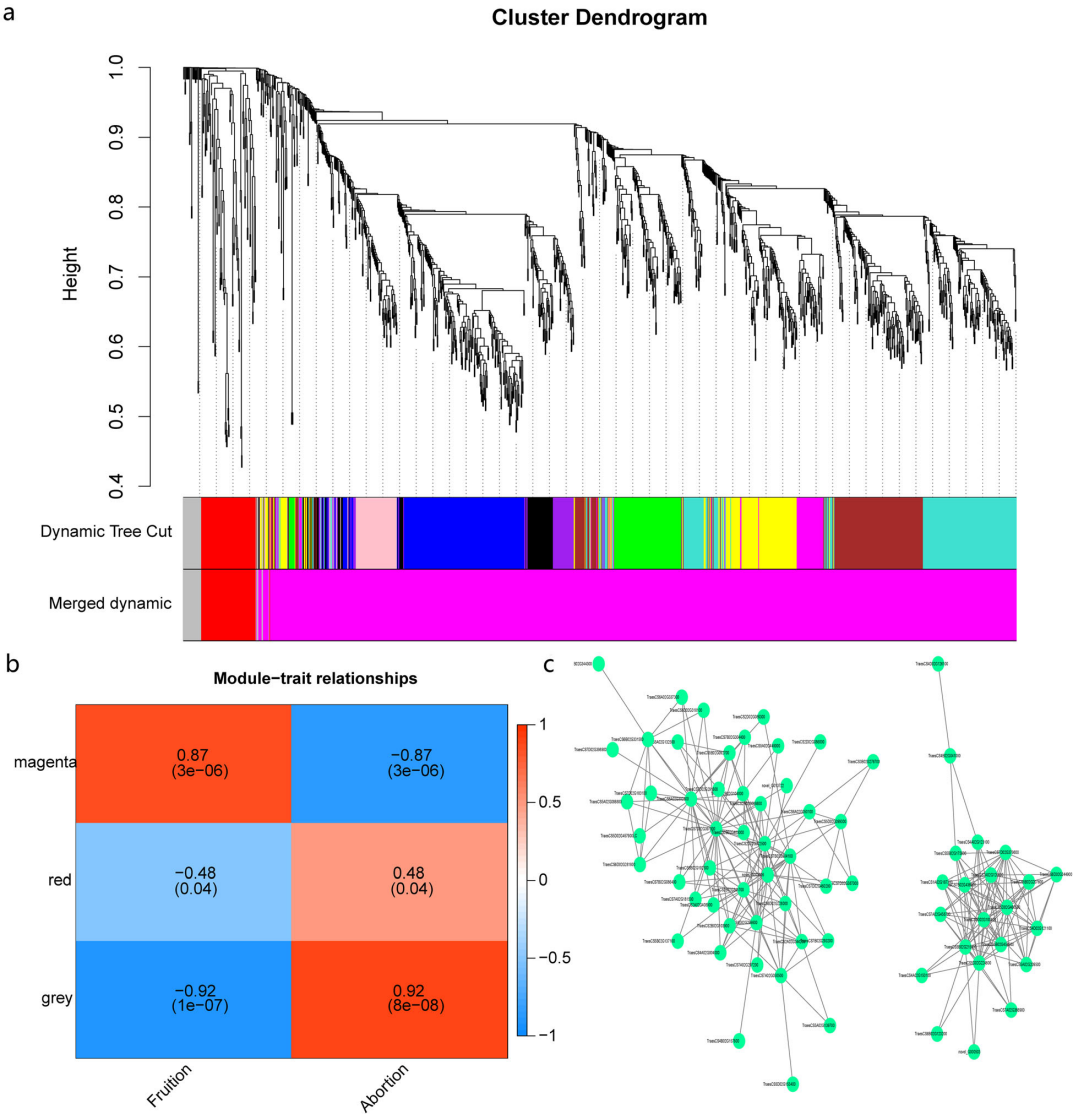
WGCNA results for core DEGs. **a** Hierarchical cluster tree showing the modules of co-expressed genes, where the lower panel shows the modules in different colors. **b** Module–trait correlations and corresponding *p*-values (in parentheses), where the left panel shows three module eigengenes (MEmagenta, MEred, and MEgrey) and the right panel shows a color scale for the module trait correlations ranging from − 1 to 1. **c** Cytoscape representation of the co-expressed genes in important pathways in the magenta module

Weighted gene co-expression network analysis (WGCNA) was performed using the non-redundant core DEGs to further elucidate the genes associated with male sterility in the five IAMSLs (Fig. 7a). Analysis of the module–trait relationships using the seed-setting percentage and pollen-abortion rate as the trait data showed that the “magenta” module (*r* = 0.87, *p* = 3e-06) was highly correlated with male sterility in the 18 samples (Fig. 7b). In addition, to further identify the important pathways in the magenta module, we conducted KEGG enrichment analysis, and found that genes in this module were mainly related to the phosphatidylinositol (PI) signaling system and three sugar metabolism pathways (pentose and glucuronate interconversions, starch and sucrose metabolism, and glycolysis/gluconeogenesis) (Additional file 1: Figure S8). The Cytoscape representation of some hub genes in the major pathways showed that the hub genes had greater connectivity, and thus they may be strongly associated with male sterility (Additional file 1: Figure S9). These hub genes included diacylglycerol kinase theta (*TraesCS7A02G297200*, *TraesCS7D02G291600*), UTP--glucose-1-phosphate uridylyltransferase (*TraesCS6A02G050100*), and pectinesterase 53 (*TraesCS5A02G086800*, *TraesCS5B02G092700, TraesCS5D02G099300*) (Fig. 7c). Importantly, we found that most of these hub genes or their homologous genes were consistent with the candidate genes mentioned above. Thus, the results also confirmed that these candidate genes are important for male sterility in wheat.

### Effects of altered gene expression in the major metabolic pathways on pollen development

**Fig. 8:**
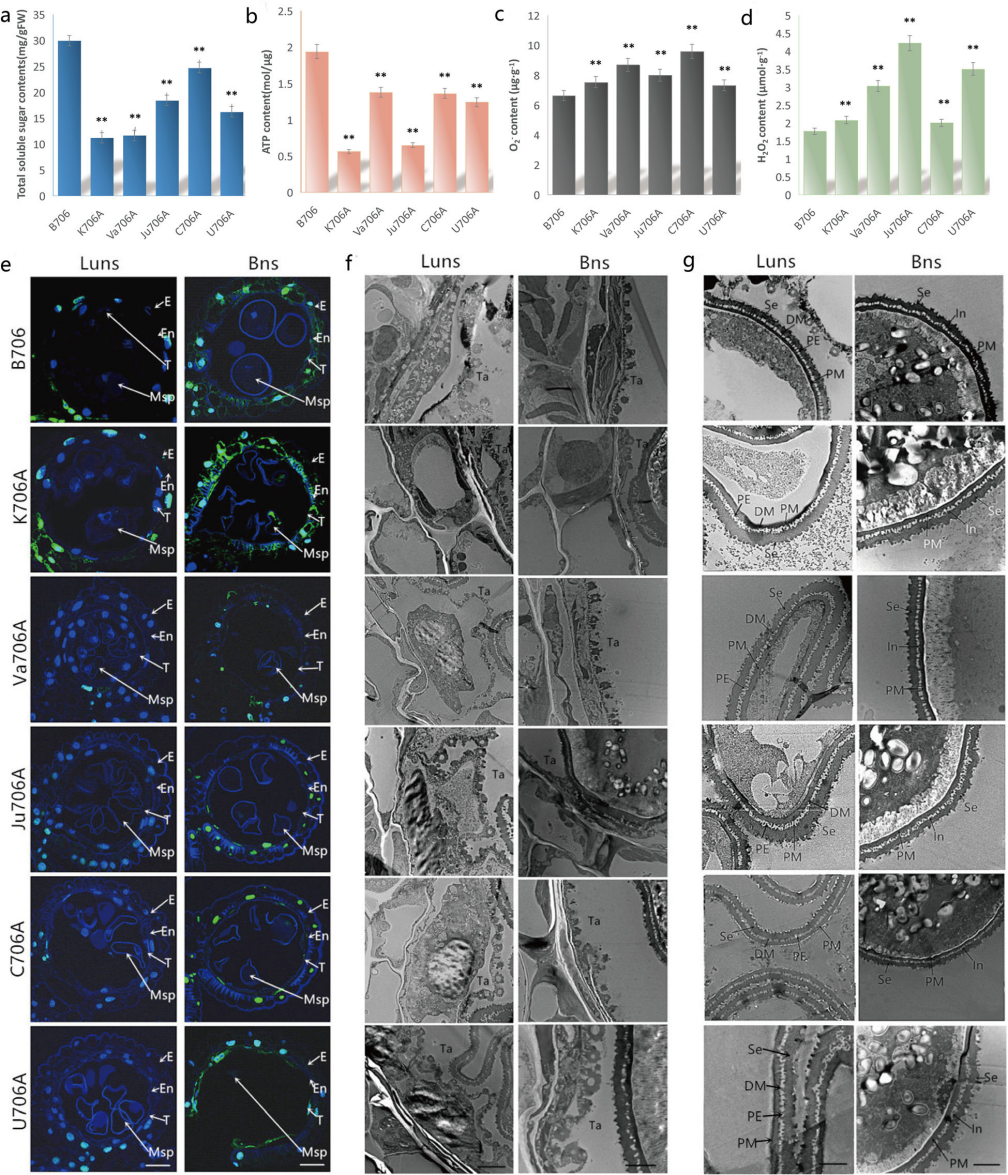
Quantification of physiological indices and histological analysis. **a** Total soluble sugar contents of anthers in the binucleate stage. **b** ATP content of anthers in the binucleate stage. **c** O_2_^−^ contents. **d** H_2_O_2_ contents. Student’s *t-*test *p < 0.05 **p < 0.01. Each value represents means ± SD (*n* = 3). **e** TUNEL assays to detect anther tapetum PCD. Scale bars represent 50 μm. **f** Transmission electron micrographs of the tapetum. Scale bars represent 2 μm. **g** Transmission electron micrographs of microspore. Scale bars represent 2 μm. Luns, late uninucleate stage; Bns, binucleate stage; E: epidermis; En: endothecium; T: tapetum; Tds: tetrads; Msp: microspores; Ta: tapetosome; PE, primexine; Se, sexine; DM, deposited materials; PM, plasma membrane; In, intine

We analyzed the total soluble sugar contents in the anthers from the IAMSLs and maintainer in the binucleate stage to verify whether the downregulated expression of these genes in sugar metabolic pathways may affect the maintenance of pollen growth and development. Compared with B706, the soluble starch contents were significantly lower in the anthers from the five IAMSLs (Fig. 8a). Thus, we suggest that soluble starch deficiency due to the limits on some genes involved in sugar metabolic pathways might be one of the main causes of pollen abortion during the later stages of pollen development in the IAMSLs.

In order to clarify the effect of down-regulated genes of oxidative phosphorylation pathway on pollen development, we measured the ATP and ROS contents in the anthers using a spectrophotometric method. We found that the ATP contents were significantly lower in the IAMSLs than B706, but excess ROS accumulated in the five IAMSLs (Fig. 8b-d). Thus, excess ROS could suppress the expression of genes related to the oxidative phosphorylation process, and lead to the availability of insufficient energy in the mitochondria.

Based on the above KEGG and hierarchical clustering analysis, our results suggest that the downregulation of these DEGs might limit the phenylpropanoid biosynthesis process. Therefore, TUNEL and transmission electron microscopy assays were conducted using anther sections from the critical stages of abortion. As shown in Fig. 8e and Additional file 1: Figure S9A, the tapetal cells produced stronger TUNEL-positive signals in K706A compared with B706 during the later uninucleate and binucleate stages. However, unlike K706A, the TUNEL fluorescence signals from other sterile lines were all weaker than those from B706 during these two periods. Furthermore, the tapetal ultrastructure assay indicated the premature apoptosis of tapetal cells in K706A during the later uninucleate stage. In contrast to K706A, the tapetum cells in the other IAMSLs were larger compared with those in the maintainer line during this stage (Figs. 8f and Additional file 1: Figure S10), which suggests that delayed PCD occurred in these tapetal cells. We also observed pollen development to determine whether the aberrant formation of sporopollenin was synchronized with the tapetal PCD defects. In contrast to B706, the abnormal accumulation of sporopollenin in the IAMSLs resulted in the inability to form normal sexine (Fig. 8g). These observations indicate that the downregulation of these DEGs related to phenylpropanoid biosynthesis might trigger abnormal PCD in the tapetum, thereby hindering the secretion of sporopollenin during pollen development to cause defective pollen exine production and male sterility in the wheat IAMSLs. These findings further confirmed our predictions regarding male sterility in wheat, and they also demonstrated that the RNA-Seq results were reliable.

### Validation of RNA-seq data by quantitative real time polymerase chain reaction 

**Fig. 9:**
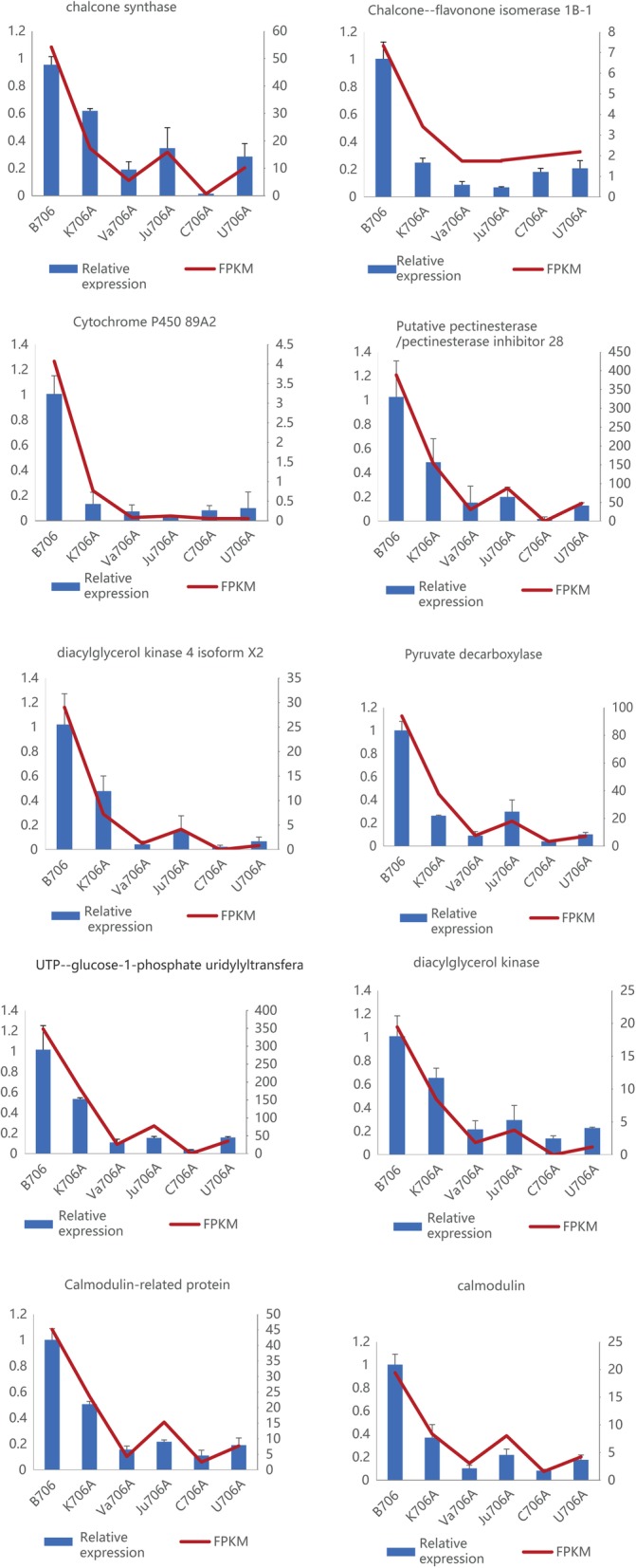
Expression levels of candidate DEGs according to RT-qPCR (blue histogram) and RNA-seq (red line chart). FPKM values are based on RNA-seq data. The data obtained by RT-qPCR are the means based on three replicates

We validated the accuracy of the RNA-seq data by conducting quantitative real time polymerase chain reaction (RT-qPCR) analysis, where 10 DEGs that had probable relationships with CMS were selected based on their functional classifications, WGCNA analysis, and the results of previous studies. The expression patterns of these candidate genes according to RT-qPCR were consistent with those determined by RNA-seq (Fig. 9). Furthermore, linear regression analysis detected positive correlations between the RT-qPCR and RNA-seq results (Additional file 1: Figure S11), thereby demonstrating that the RNA-Seq results were accurate and reliable in this study.

## Discussion

### DEGs involved in sugar metabolism related to CMS

In plants, sucrose and starch are accumulated as energy reserves and carbon skeleton sources, and thus they are necessary for pollen and anther development in the later mature developmental stages by providing basic materials for constructing plant cell walls and fibers. Pentose and glucuronate interconversions, starch and sucrose metabolism, and glycolysis/gluconeogenesis are important sugar metabolism pathways during anther development, and the disruption of sugar metabolism can severely damage pollen development to eventually cause male sterility. In general, the pollen will mature early if the starch level is kept below a certain threshold [30]. For example, in male sterility CMS mutants, the lack of pollen viability is closely related to starch deficiency [31]. Ma et al. [32] employed transcriptome sequencing to identify 1165 DEGs enriched during anther development and many were related to starch and sucrose metabolism, pentose glucuronic acid transformation, and flavonoid biosynthesis metabolism. Other studies indicate that glucose metabolism pathways, such as pentose glucuronic acid transformation, starch and sucrose metabolism, and galactose metabolism, are essential for anther development in cotton. The key genes required for these regulatory pathways may be involved in genetic male sterility anther development [33]. In the present study, 23 DEGs were downregulated in all of the IAMSLs, including three in important sugar metabolism pathways. In particular, among these DEGs, pyruvate decarboxylase (PDC) is a carboxyl lyase where thiamine pyrophosphate (TPP) is its coenzyme, and it is widespread in plants. As a key enzyme in the fermentation pathway. By competing with pyruvate dehydrogenase, pyruvic acid participates in energy and material metabolism in plants to play important roles in the development of nodule tissue, the release of dormant buds, fruit ripening, pollen development, and pollen tube germination [34]. In addition, we found that the DEG encoding UTP--glucose-1-phosphate uridylyltransferase, such as UGP1, was strongly downregulated in IAMSLs. The UGPases in plants have four main roles: participating in the synthesis and metabolism of sucrose, plant cell wall synthesis, and the reproductive process [19]. Thus, the DEG expression patterns determined in our study suggest that the consumption of sugar was accelerated in the anthers and sucrose transport was reduced to lead to sterility.

### DEGs involved in PI signaling system related to CMS

The plasma membrane is an important structure for maintaining cell viability. PI is one of the major phospholipids in the cell membrane and a major phospholipid signaling molecule because of its special molecular structure, where the PI signal transduction process has many important roles [35]. PI-specific phospholipase C, a key enzyme in the PI signaling system, which is activated by receptors. When cells are stimulated by external factors, PI 4,5-bisphosphate produces the messenger molecules inositol 1,4,5-trisphosphate and diacylglycerol, which regulate the concentration of Ca^2+^ and the activity of protein kinase C in the cells [36]. The PI signaling system also mediates the transmembrane transduction of extracellular signals to play important roles in the regulation of plant growth and development, and the responses to environmental stimuli. Pollen development comprises a series of processes involving meiosis and differentiation, including dynamic changes in the cytoplasmic components and internal subcellular components, with changes in the cytoskeleton and vacuoles. Pollen tube growth also depends on cytoskeletal reorganization and vesicle transport [37]. Vesicle transport allows the pollen tube to coordinate the balance between the cell components and plasma membrane, and to control the polar growth process effectively. Many studies have shown that various components of the PI signaling system are involved in vacuolar changes during pollen development and vesicle transport in pollen tube growth [38]. When the expression of these enzymes is abnormal, the vesicle transport process may be affected in the pollen tube to prevent pollen germination and lead to pollen abortion. Thus, the downregulated genes related to PI signaling system detected in the present study may have been main cause of male sterility.

### DEGs involved in oxidative phosphorylation and phenylpropanoid biosynthesis related to CMS

Mitochondria are important sites of cellular metabolism and energy metabolism [39]. Various enzymes located in mitochondria are responsible for the decomposition and release of energy in the respiration process. In addition, except for the transformation of some energy into heat, most of the energy released by respiration is stored in the form of ATP to provide the energy for life activities and the growth and development of plants [40]. Under normal conditions, substrates such as succinic acid and NADH are often sufficient for respiratory electron transport, but the supply of ADP is considered a limiting factor that regulates the overall respiratory process. Hence, when the electron transport chain is suppressed, electrons will interact directly with oxygen molecules to produce ROS, and this excess ROS will lead to abnormal PCD in the tapetum and male sterility [41, 42]. Phenylpropane metabolism has two important branches comprising the lignin metabolic pathway and flavonoid metabolic pathway. P-coumaric acid coenzyme A (CoA) enters the lignin synthesis under the action of hydroxyl cinnamyl transferase (HCT). Subsequently, 11 different lignocellulosic monomers are generated through a series of hydroxylation, methylation, linkage, and reduction reactions. These lignocellulosic monomers are synthesized in the cytoplasm and then transported to the cell wall, where they are polymerized into lignin. P-coumaric acid CoA and propane two acyl CoA enter the branching flavonoid synthesis pathway under the action of chalcone synthetase [43]. Flavonoids are important constituents of anthocyanin and they exist in the outer wall of the anther. Phenylalanine ammonia lyase, cinnamic acid hydroxylase, and other key phenylpropane metabolism enzymes are synthesized and secreted by tapetum cells into the outer wall of pollen, where they participate in phenylpropane metabolism in the outer chamber of the developing microspores to finally form sporopollenin [26]. Thus, in our study, the upregulation of genes related to oxidative phosphorylation may been caused by the effects of excessive ROS accumulation on the electron transport chain, where the excess ROS acted as a molecular signal to downregulate phenylpropanoid biosynthesis genes and contribute to abnormal tapetal PCD.

### Possible transcriptome-mediated male sterility network in wheat

**Fig. 10:**
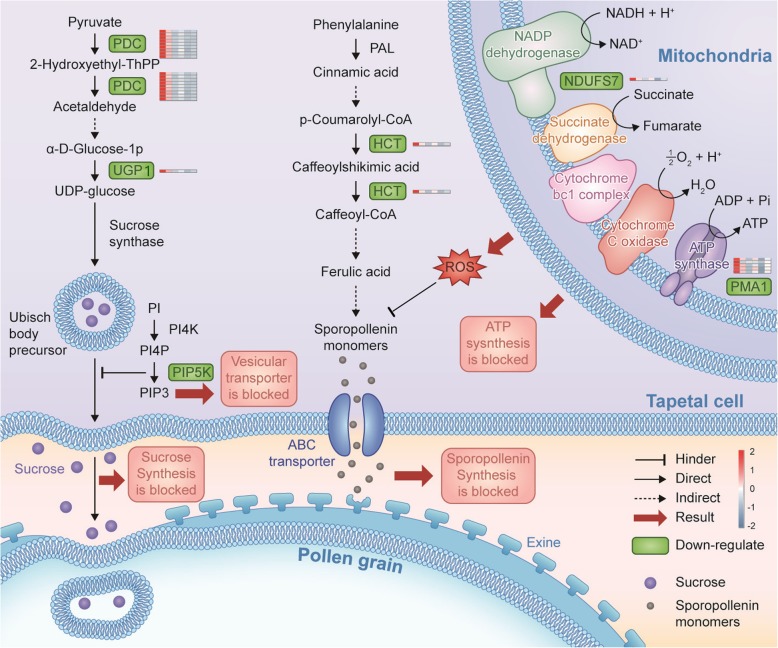
Possible transcriptome-mediated male sterility network in wheat. PDC, pyruvate decarboxylase; UGP1, UTP-glucose-1-phosphate uridylyltransferase; PIP5K, 1-phosphatidylinositol-4-phosphate 5-kinase; HCT, hydroxyl cinnamyl transferase; NDUFS7, NADH dehydrogenase; PMA1, ATPase. We used the software of Adobe Illustrator CS5 (Adobe, America) to draw this figure by ourselves

According to previously published results and based on the putative functions and changes in the expression patterns of 1,281 DEGs and their experimental verification in the present study, we propose an intriguing transcriptome-mediated male sterility network for wheat, as shown in Fig. 10. This network has several functional components comprising the inhibited synthesis and transport of sucrose, blocked synthesis of sporopollenin, accelerated generation of ROS, cell wall disruption, and abnormal tapetal PCD. The tapetum comprises the innermost layer of anther cells, which provides sucrose, proteins, lipids, and sporopollenin to support the growth and development of the microspore via its degradation and secretion [44]. Pyruvate decarboxylase (PDC) and UGP participate in the synthesis of sucrose and sugar metabolism in plants, which are essential for pollen development and cell wall formation [45]. As shown in Fig. 9, compared with the maintainer, the expression levels of the genes encoding PDC and UGP were greatly downregulated, and the total soluble sugar contents were reduced in the IAMSLs. Sucrose is transported from the tapetum to the microspore via vesicle transport [46]. Moreover, 1-PI-4-phosphate 5-kinase (PIP5K) is a key enzyme in vesicle transport and it participates in the PI signaling system pathway. Thus, downregulation of the PIP5K gene will affect the transportation of sucrose to the microspore, thereby hindering pollen wall synthesis [37]. In the present study, genes related to NADH dehydrogenase and ATP synthase were greatly downregulated in the IAMSLs, and these abnormal changes led to superabundant electrons binding directly to molecular oxygen to generate excess ROS and reducing ATP production in the IAMSLs. Excess ROS results in membrane lipid peroxidation and severely increases the permeability of cells [20]. HCT is involved in the phenylpropanoid biosynthesis pathway and the sporopollenin synthesis process [47]. The downregulation of HCT-related genes and excessive accumulation of ROS may have to the blockage of sporopollenin synthesis, thereby ultimately affecting the formation of the microspore outer wall. Thus, the combined effect of these changes led to pollen abortion in the IAMSLs.

## Conclusions

Based on our results, during the vital abortive stage, we found most of the 1281 core shared DEGs were downregulated in the IAMSLs. Furthermore, the key candidate genes related to male sterility were screened and identified, which encoding glycolipid transfer protein, pectinesterase, and UDP-glucose pyrophosphorylase. Moreover, according to our bioinformatics analysis and further verification, we proposed an intriguing core transcriptome-mediated male-sterility network for wheat, and inferred that due to the downregulation of genes in sugars, oxidative phosphorylation, phenylpropane biosynthesis, and phosphatidylinositol signaling pathways, the sugar and energy of anthers were deficient, with excessive accumulations of ROS, thereby triggering the abnormal tapetum degradation and blocked sporopollenin synthesis. These results provide new insights into the metabolic pathways involved with anther abortion in CMS.

## Methods

### Plant materials

Five types of IAMSLs were used in this study, i.e., K706A with *Aegilops kotschyi* cytoplasm, Va706A with *Ae. vavilovii* cytoplasm, Ju706A with *Ae. juvenalis* cytoplasm, C706A with *Ae. crassa* cytoplasm, and U706A with *Ae. uniaristata* cytoplasm, and their maintainer line B706 as a control, where they all had the same nuclear background (All of materials were developed by our research group, K-type wheat hybrid breeding program, at Northwest A&F University, China.). The sterile lines were obtained from stable sterile lines by backcrossing over 20 times with B706. To confirm the stability of sterility, the five IAMSLs were planted during October 2015 in wheat experimental fields at Northwest A&F University, Yangling, China (108°E, 34°15′N). During April 2016, each IAMSL was checked by bagging and the self-setting rates of the lines were all zero, thereby indicating that male sterility was stable and confirming previous findings [5]. We recorded the dates for the major growth stages and young panicle differentiation stages in the five types of IAMSLs and their maintainer line B706. The overall anther development period was divided into five stages based on the progress of microsporogenesis: tetrad stage, early uninucleate stage, late uninucleate stage, binucleate stage, and trinucleate stage [41]. In order to ensure the reliability of the RNA-seq results, a randomized complete block design was used with three biological replications, where anthers with the same length (in the binucleate stage) were mixed separately from 50 plants for each biological replicate of each material, snap frozen in liquid nitrogen, and stored at − 80 °C until RNA extraction and physiological index verification. Moreover, anthers from the five IAMSLs and their maintainer line were also harvested in the five stages for cytological experiments.

### Phenotypic characterization of IAMSLs and their maintainer line

The stages of microspore development were identified by staining the nuclei with acetic acid magenta and microscopy, as described by Ba et al [48]. Anthers from the trinucleate stage were photographed using a Motic K400 stereomicroscope (Hong Kong, China). Dehiscent anthers from mature flowers were stained with I_2_-KI to evaluate the viability of mature pollen grains [49]. Anthers and microspores were collected in the trinucleate stage, before fixing in 2.5% glutaraldehyde, dehydrating using a graded ethanol series (50% twice, 60, 70, 80, 90, 95, and 100% twice), dried [50], and imaging by scanning electron microscopy (JEOL JSM-6360LV). Morphological observations of the pollen grains and chromosomes were made in different microspore developmental stages by staining the nuclei with DAPI, before photographing with a DS-U2 high resolution camera mounted on a Nikon ECLIPSE E600 microscope (Nikon Corporation, Japan) and processing with NIS-Elements software.

### RNA extraction, and cDNA library construction and sequencing

Total RNA from each of 18 samples (three biological replications of each material in the binucleate stage) was isolated using an RNAiso for Polysaccharide-rich Plant Tissue kit (Takara Biological Engineering (Dalian) Co. Ltd., China) according to the instructions provided by the manufacturer. For each RNA sample, the concentration was measured with a NanoDrop 2000 spectrophotometer (NanoDrop Technologies Inc., USA). The integrity of RNA was confirmed using RNase-free agarose gel electrophoresis, before measuring the concentration with a 2100 Bioanalyzer (Agilent Technologies, Santa Clara, CA). mRNA enrichment and purification were conducted using 5 μg of total RNA with oligo (dT)-attached magnetic beads, before breaking into short fragments. The cleaved mRNA fragments were used as templates for first-strand cDNA synthesis with random hexamers and SuperScript II. Second-strand cDNA was synthesized with DNA polymerase I, dNTPs, and RNaseH. The cDNA fragments were then subjected to end-repair, before adding poly-(A) tails together with a ligation sequencing adapter, and purifying the fragments, which were then selected by size with AMPure XP beads. Subsequently, PCR amplification was employed for enrichment of the purified cDNA templates. Finally, Illumina sequencing was performed with the Illumina HiSeq PE150 platform by Guangzhou Sagene Biotechnology Co. Ltd. (Guangzhou, China).

### Data processing and identification of DEGs

In order to obtain high-quality clean reads (clean data) for sequence analysis, adaptor sequences, low-quality reads (base number SQ ≤ 20 in more than 50% of the whole reads), and reads with over 10% unknown bases (N bases) were removed [51]. The high-quality clean reads were then mapped to the ribosomal database (ftp://ftp.ncbi.nlm.nih.gov/genbank) and the mapped reads were removed. Next, based on the wheat reference genome (IWGSC1.0 + NC_002762.1, http://plant.ensembl.org/index.html), all of the high-quality clean reads excluding rRNA were mapped using HISAT2 (https://ccb.jhu.edu/software/hisat2/index.shtml). The mapped clean reads were assembled with StringTie (https://ccb.jhu.edu/software/stringtie/index.shtml) [52]. The expression level of each gene was quantified using RESM (v1.2.31) and the DEGs were calculated using DESeq2, *p*-adjust < 0.05 [53].

### Functional annotations and DEG analysis

The shared DEGs in the IAMSLs were functionally classified using BLASTx 2.2.24+ software with the STRING9.0 database (http://string-db.org/) based on the KOGs database (ftp://ftp.ncbi.nih.gov/pub/COG/KOG/). GO (http://www.geneontology.org/) and KEGG (http://www.genome.jp/kegg/) functional annotations for the shared DEGs were obtained using BLAST2GO (http://www.blast2go.com/) and BLASTx/BLASTp 2.2.24+, respectively. GO term and KEGG pathway enrichment analysis were performed using the shared DEGs with Goatools and KOBAS software, where the threshold was a corrected *P*-value ≤0.05 [54]. The relationships between the key GO terms and associated DEGs were visualized using Circos version 0.69 [55]. The heatmap was prepared using Ggplot in R.

### Co-expression network construction

WGCNA was performed for the shared DEGs by using WGCNA in the R package to infer the co-expressed gene modules that had strong associations with sterility [56]. The adjacency matrix was obtained based on the pairwise Pearson’s correlation coefficients between pairs of genes. Construction of the WGCNA network and module testing were performed using an unsigned type topology overlap matrix, with a power β of 13 and a branch merge cut height of 0.25. The module eigengene (the first principal component of a given module) was calculated and used to evaluate the module’s correlation with sterility. The interaction network diagram was conducted according to KEGG pathway enrichment analysis by using Cytoscape 3.1.1 to visualize the key modules and DEGs [57].

### Sugar, ATP, and reactive oxygen species (ROS) contents

Anthers (0.2 g) were collected during the binucleate stage from each material to determine the soluble sugar (sucrose, fructose, and glucose) and ATP contents. The total sugar contents and ATP contents were measured at 620 nm and 700 nm using an ultraviolet spectrophotometer (Philes, Nanjing, China, http://www.philes.cn/) with a Soluble Sugars Assay kit and an ATP Detection Kit (Comin, Suzhou, China, http://www.cominbio.com/), respectively, according to the instructions supplied with the kits. The total sugar contents (mg g^− 1^) and ATP contents (μmol g^− 1^) were calculated based on the formulae provided with the instructions. The rate of superoxide anion (O_2_^−^) production and the H_2_O_2_ contents were measured using 0.5 g of anthers from the IAMSLs and B706 as described previously [58]. All measurements of physiological indexes were conducted based on three biological replicates.

### TUNEL assay

Paraffin sections of anthers (6 μm) were dehydrated using a graded ethanol series and incubated in 20 μg L^− 1^ Proteinase K (Roche) for 10 min, before washing with phosphate-buffered saline for 15 min. TUNEL assays were conducted with an In Situ Cell Death Detection Kit (POD, Roche) according to the manufacturer’s instructions. The sections were then observed with a fluorescence confocal scanning microscope (Nikon, Tokyo, Japan). The green TUNEL fluorescent signal and blue DAPI fluorescence were analyzed at excitation and emission wavelengths of 450 nm and 515 nm and 358 nm and 461 nm, respectively. In addition, we counted the TUNEL-positive nuclei/total nuclei ratio in the key stages using IPP 6.0 software.

### Transmission electron microscopy observations

To obtain ultrathin sections, anthers from the late uninucleate and binucleate stages were fixed in 2.5% glutaraldehyde for > 24 h at 4 °C. After washing with phosphate buffer (0.1 mol L^− 1^, pH 7.2), the anthers were fixed in 1% osmium tetroxide and dehydrated with an ethanol series (50% twice, 60, 70, 80, 90, 95, and 100% twice). The anthers were prepared with a UC6 ultramicrotome (Leica) and stained using 0.2% lead citrate [44]. The ultrastructure was observed for the tapetum and microspore using a transmission electron microscope (Hitachi, H-7650, Japan) [59]. The area of the tapetum in each section was calculated using CellSens Entry software.

### Quantitative RT-PCR (RT-qPCR) validation

The synthesis of first-strand cDNA was conducted using 5 μg of total RNA from each material with a Transcriptor First Strand cDNA Synthesis Kit (Roche, Germany) according to the manufacturer’s instructions. The *TaActin* gene was employed as an internal reference gene for RT-qPCR [60]. The primers for the selected candidate genes and *TaActin* gene were designed using Primer Premier 5.0, and they are listed in Additional file 2: Table S1. Reactions were performed using 2× RealStar SYBR Mixture (with ROX II; GenStar, China) in the QuantStudio™ 7 Flex Real-Time PCR System (Applied Biosystems, USA) according to the following program: 95 °C for 10 min, followed by 40 cycles at 95 °C for 15 s and 60 °C for 1 min. RT-qPCR was conducted in triplicate (technical repeats) with three biological replicates for each material, and the relative mRNA levels were calculated using the 2^–ΔΔCt^ method [61].

### Statistical analysis

Statistical analyses were performed for the measurements of physiological indexes results using one-way analysis of variance. Significant differences in the expression levels between the IAMSLs and B706 were evaluated with the Duncan test (* *p* < 0.05; ** *p* < 0.01) using SPSS statistical software and Excel Office.

## Supplementary information

**Additional file 1 **: **Figure S1.** Comparisons of the stamens and pistils and I_2_-KI staining in the IAMSLs and B706 at the trinucleate stage. A and G, B706; B and H, K706A; C and I, Va706A; D and J, Ju706A; E and K, C706A and F and L, U706A. A to F: anthers. G to L: microspores by I_2_-KI staining. Scale bars represent 0.5 mm. **Figure S2.** Number and percentage analysis of genes at different expression levels. The FPKM value of gene expression was divided into 4 grades: 0~1, 1~5, 5~30, and above 30, and the number and percentage of genes in each grade were counted. **Figure S3.** FPKM violin distribution analysis. The horizontal axis indicates different samples, and the vertical axis indicates corresponding sample FPKM. **Figure S4.** Correlation analysis between biological replicates. The horizontal axis and vertical axis represent each sample. The color represents the correlation coefficient, the redder the color, the higher the correlation, and the whiter the color, the lower the correlation. **Figure S5.** Histogram of clusters of Eukaryotic Orthologous Groups (KOG) classification. Abscissa for the number of genes, Ordinate for each KOG classified content. **Figure S6.** GO annotation classification of the core differentially expressed genes. **Figure S7.** GO enrichment analysis of the core differentially expressed genes. (A) cellular component GO categories enrichment analysis; (B) molecular function GO categories enrichment analysis; (C) biological process GO categories enrichment analysis. **Figure S8.** Bubble diagram for KEGG enrichment in the DEGs of magenta module. **Figure S9.** Cytoscape representation of co-expressed genes in the magenta module. **Figure S10.** Sectional area of the tapetum cell (A) and TUNEL positive nuclei/total nuclei proportion for key stages (B). **Figure S11.** Coefficient analysis of fold change data between RT-qPCR and RNA-seq. Data indicating relative transcript level from RT-qPCR are means of three replicates. Scatterplots were generated by log2 expression ration from RNA-seq (x-axis) and RT-qPCR (y-axis).

**Additional file 2: Table S1.** Sequence-specific primers used for RT-qPCR.

**Additional file 3: Table S2.** Summary and evaluation of sequencing results.

**Additional file 4: Table S3.** Annotation and FPKM of 1281 core genes.

## Data Availability

All of datasets supporting the conclusions of this article are included within the article (and its Additional files). The raw data in this study have been deposited in the Genome Sequence Archive (Genomics, Proteomics & Bioinformatics 2017) in BIG Data Center (Nucleic Acids Res 2019), Beijing Institute of Genomics (BIG), Chinese Academy of Sciences, under accession numbers CRA002161, CRA002161 that are accessible at https://bigd.big.ac.cn/gsa/browse/CRA002161.

## Abbreviations

Bns: binucleate stage
CMS: cytoplasmic male sterility
CoA: P-coumaric acid coenzyme A
DAPI: 4’,6-diamidino-2-phenylindole
DEGs: differentially expressed genes
Euns: early uninucleate stage
FPKM: fragments per kilobase of exon model per million reads mapped
GO: Gene Ontology;
HCT: hydroxyl cinnamyl transferase
I_2_–KI: iodine-potassium iodide
IAMSLs: isonuclear alloplasmic male sterile lines
KEGG: Kyoto Encyclopedia of Genes and Genomes
KOG: Eukaryotic Orthologous Groups
Luns: late uninucleate stage
PCD: programmed cell death
PDC: pyruvate decarboxylase
PI: phosphatidylinositol
PIP5K: 1-PI-4-phosphate 5-kinase
ROS: reactive oxygen species
SEM: scanning electron microscopy
Tds: tetrads
TEM: transmission electron microscopy
Tns: trinucleate stage
TPP: thiamine pyrophosphate
TUNEL: terminal deoxynucleotidyl transferase-mediated dUTP nick-end labeling
Uby: Ubisch bodies
UGP1: UTP--glucose-1-phosphate uridylyltransferase 1
UGP2: UTP--glucose-1-phosphate uridylyltransferase 2
WGCNA: weighted gene co-expression network analysis
