# Supplementary material for: Comparative transcriptome analysis indicates that a core transcriptional network mediates isonuclear alloplasmic male sterility in wheat (Triticum aestivum L.)
Source: BMC Plant Biol. 2020 Jan 7;20:10. doi: 10.1186/s12870-019-2196-x (PMC6947873; doi:10.1186/s12870-019-2196-x)
Supplement: Supplementary file 1 — Additional file 1 : Figure S1. Comparisons of the stamens and pistils and I2-KI staining in the IAMSLs and B706 at the trinucleate stage. A and G, B706; B and H, K706A; C and I, Va706A; D and J, Ju706A; E and K, C706A and F and L, U706A. A to F: anthers. G to L: microspores by I2-KI staining. Scale bars represent 0.5 mm. Figure S2. Number and percentage analysis of genes at different expression levels. The FPKM value of gene expression was divided into 4 grades: 0~1, 1~5, 5~30, and above 30, and the number and percentage of genes in each grade were counted. Figure S3. FPKM violin distribution analysis. The horizontal axis indicates different samples, and the vertical axis indicates corresponding sample FPKM. Figure S4. Correlation analysis between biological replicates. The horizontal axis and vertical axis represent each sample. The color represents the correlation coefficient, the redder the color, the higher the correlation, and the whiter the color, the lower the correlation. Figure S5. Histogram of clusters of Eukaryotic Orthologous Groups (KOG) classification. Abscissa for the number of genes, Ordinate for each KOG classified content. Figure S6. GO annotation classification of the core differentially expressed genes. Figure S7. GO enrichment analysis of the core differentially expressed genes. (A) cellular component GO categories enrichment analysis; (B) molecular function GO categories enrichment analysis; (C) biological process GO categories enrichment analysis. Figure S8. Bubble diagram for KEGG enrichment in the DEGs of magenta module. Figure S9. Cytoscape representation of co-expressed genes in the magenta module. Figure S10. Sectional area of the tapetum cell (A) and TUNEL positive nuclei/total nuclei proportion for key stages (B). Figure S11. Coefficient analysis of fold change data between RT-qPCR and RNA-seq. Data indicating relative transcript level from RT-qPCR are means of three replicates. Scatterplots were generated by log2 expression ration from [file 12870_2019_2196_MOESM1_ESM.docx]

**Additional files**

**Additional file 1: Figure S1.** Comparisons of the stamens and pistils and I_2_-KI staining in the IAMSLs and B706 at the trinucleate stage. A and G, B706; B and H, K706A; C and I, Va706A; D and J, Ju706A; E and K, C706A and F and L, U706A. A to F: anthers. G to L: microspores by I_2_-KI staining. Scale bars represent 0.5 mm.


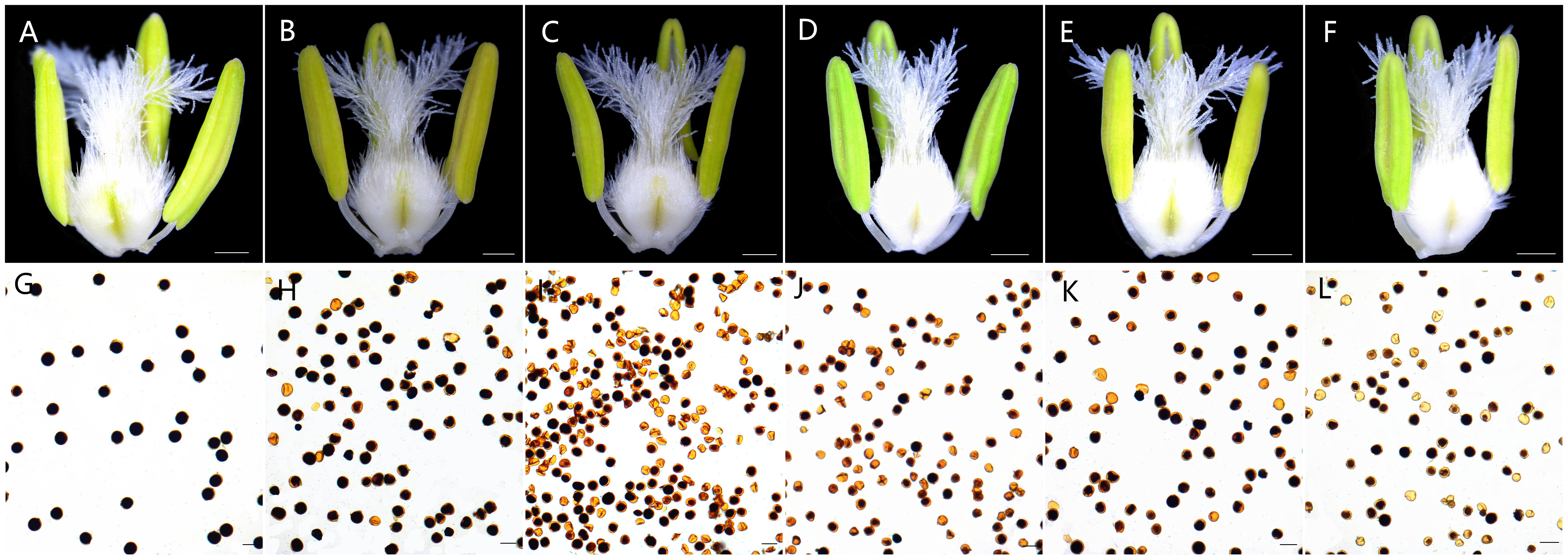


**Additional file 2: Figure S2.** Number and percentage analysis of genes at different expression levels. The FPKM value of gene expression was divided into 4 grades: 0~1, 1~5, 5~30, and above 30, and the number and percentage of genes in each grade were counted.





**Additional file 3: Figure S3.** FPKM violin distribution analysis. The horizontal axis indicates different samples, and the vertical axis indicates corresponding sample FPKM.


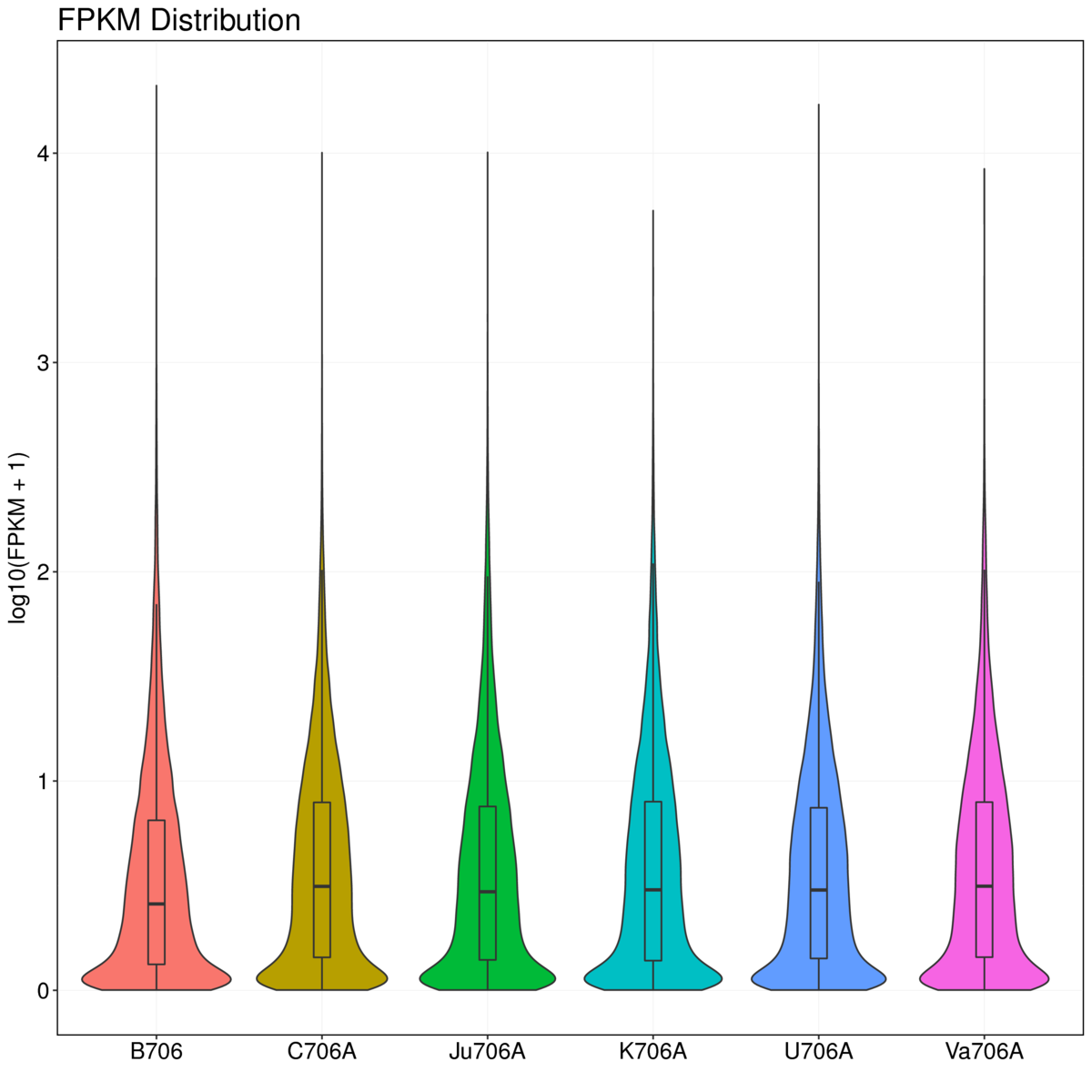


**Additional file 4: Figure S4.** Correlation analysis between biological replicates. The horizontal axis and vertical axis represent each sample. The color represents the correlation coefficient, the redder the color, the higher the correlation, and the whiter the color, the lower the correlation.


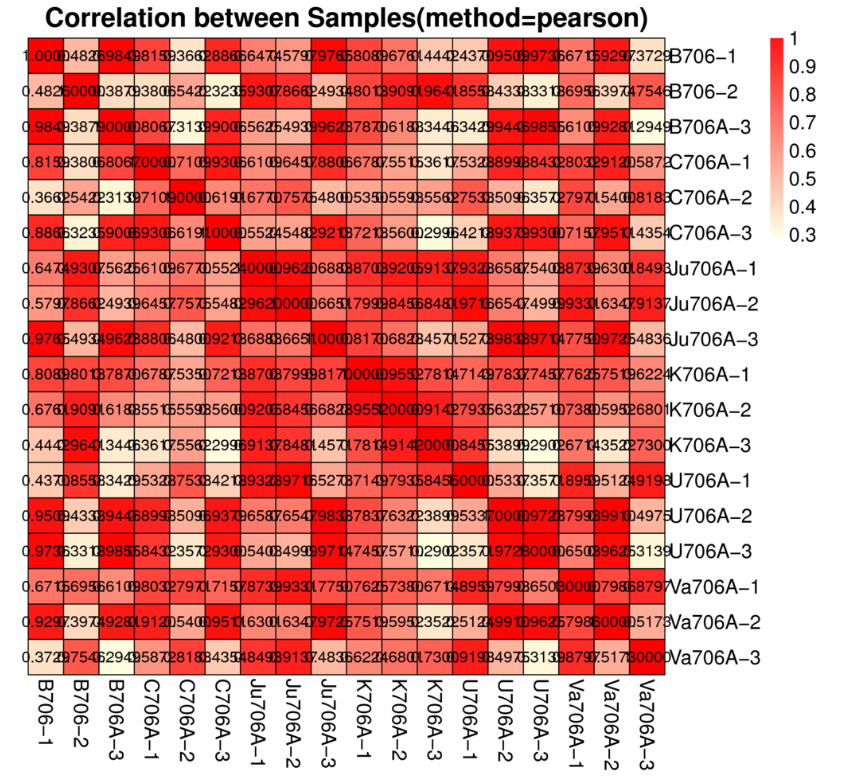


**Additional file 5: Figure S5.** Histogram of clusters of Eukaryotic Orthologous Groups (KOG) classification. Abscissa for the number of genes, Ordinate for each KOG classified content.


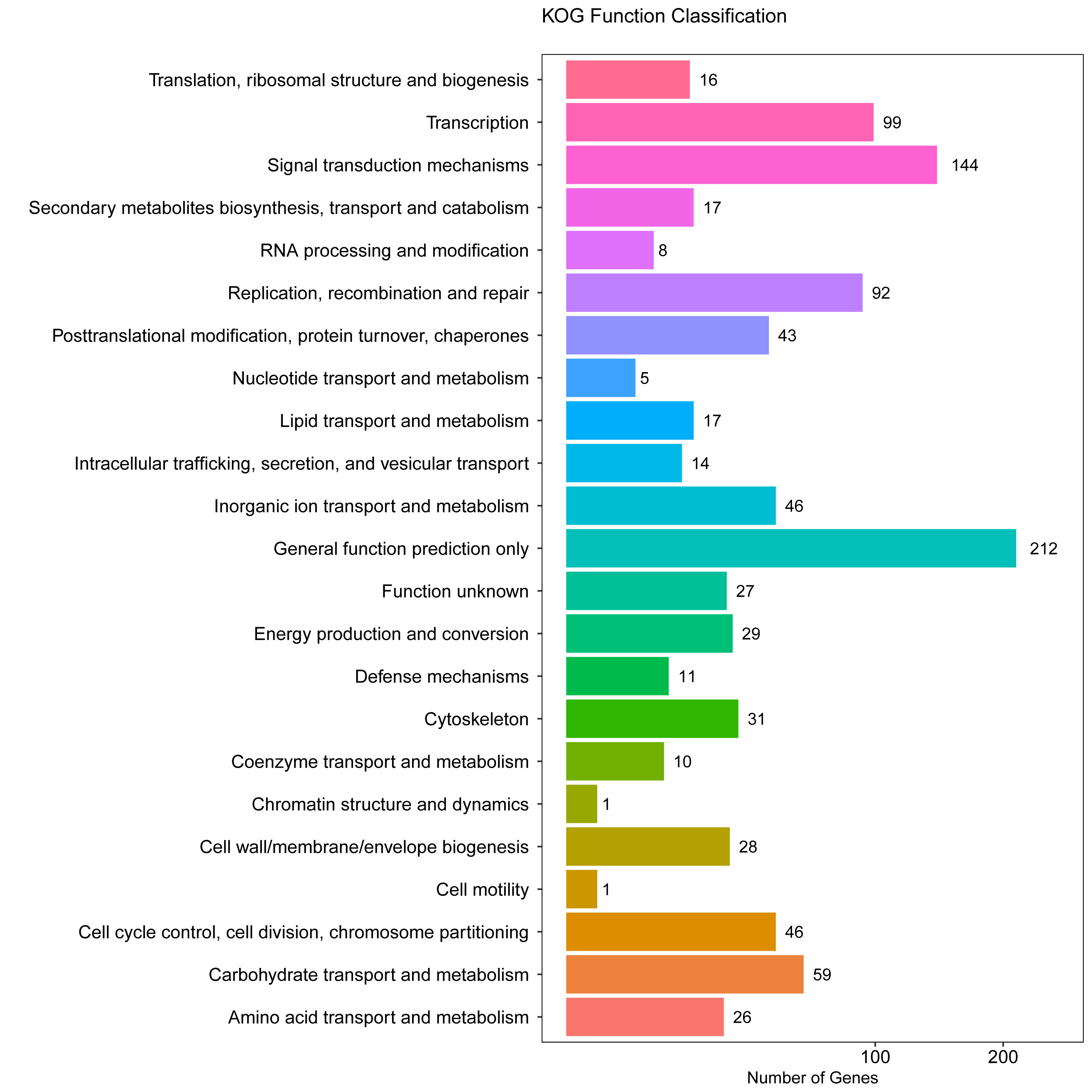


**Additional file 6: Figure S6.** GO annotation classification of the core differentially expressed genes.


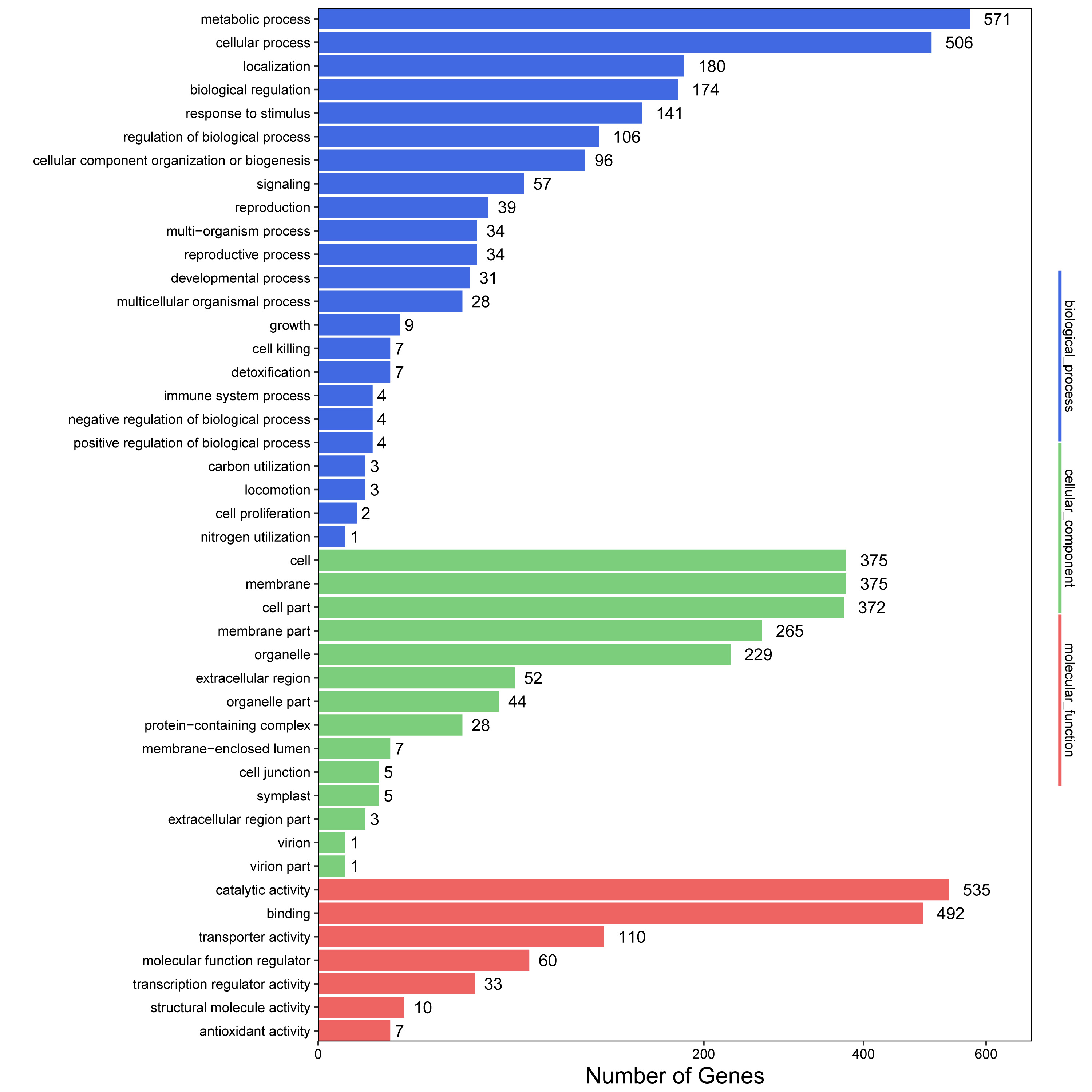


**Additional file 7: Figure S7.** GO enrichment analysis of the core differentially expressed genes. (A) cellular component GO categories enrichment analysis; (B) molecular function GO categories enrichment analysis; (C) biological process GO categories enrichment analysis.


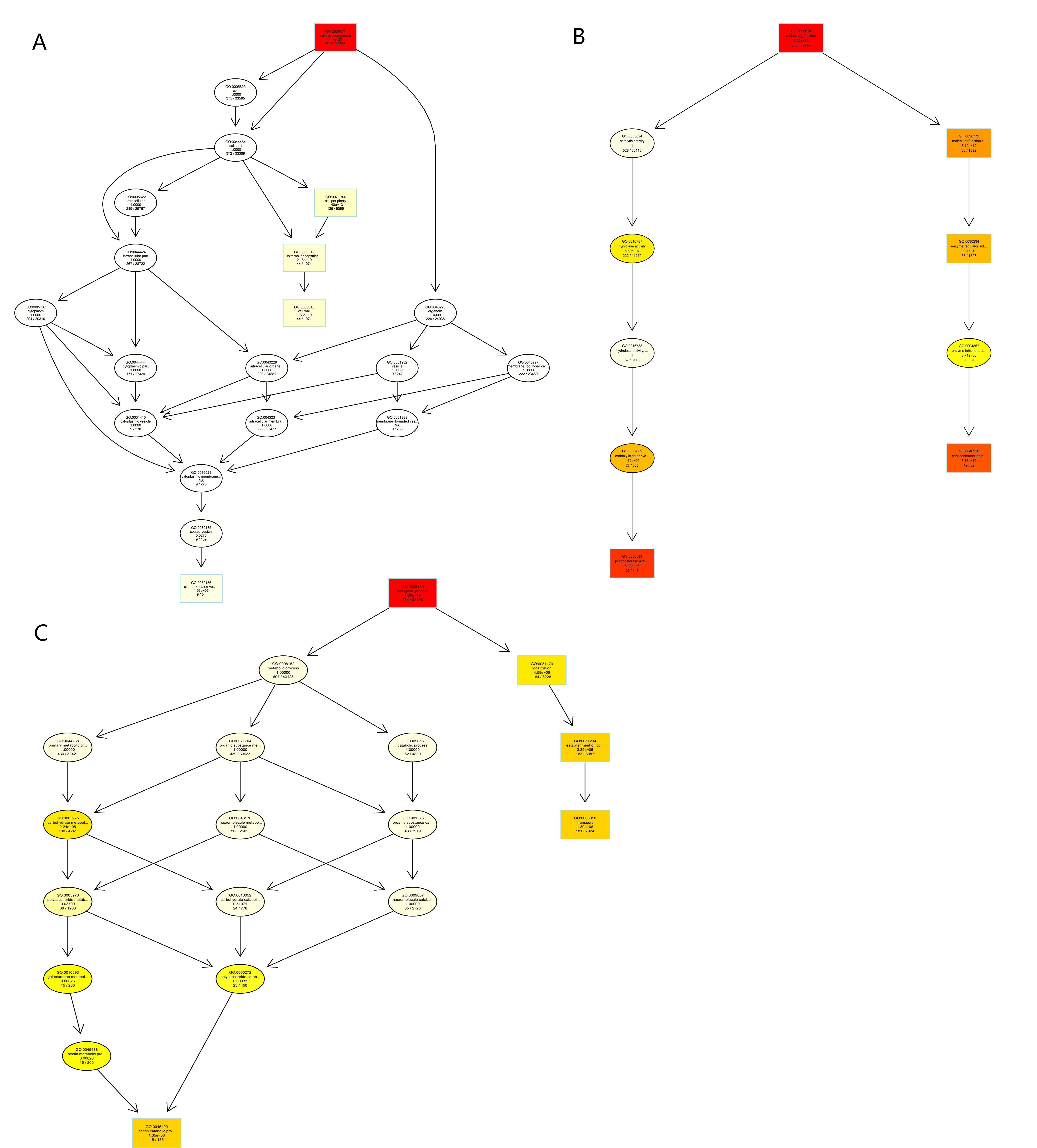


**Additional file 8: Figure S8.** Bubble diagram for KEGG enrichment in the DEGs of magenta module.

**
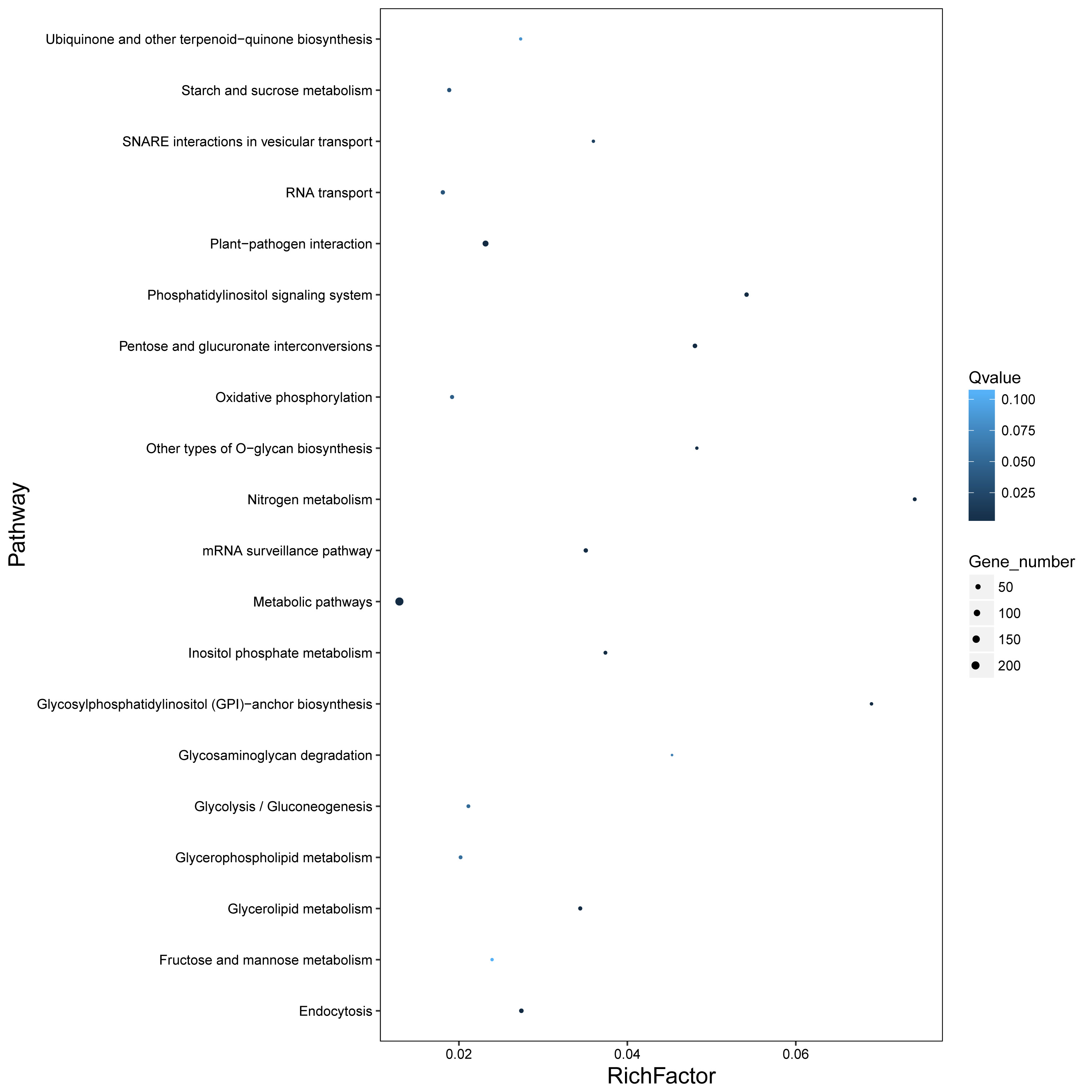
**

**Additional file 9: Figure S9.** Cytoscape representation of co-expressed genes in the magenta module.


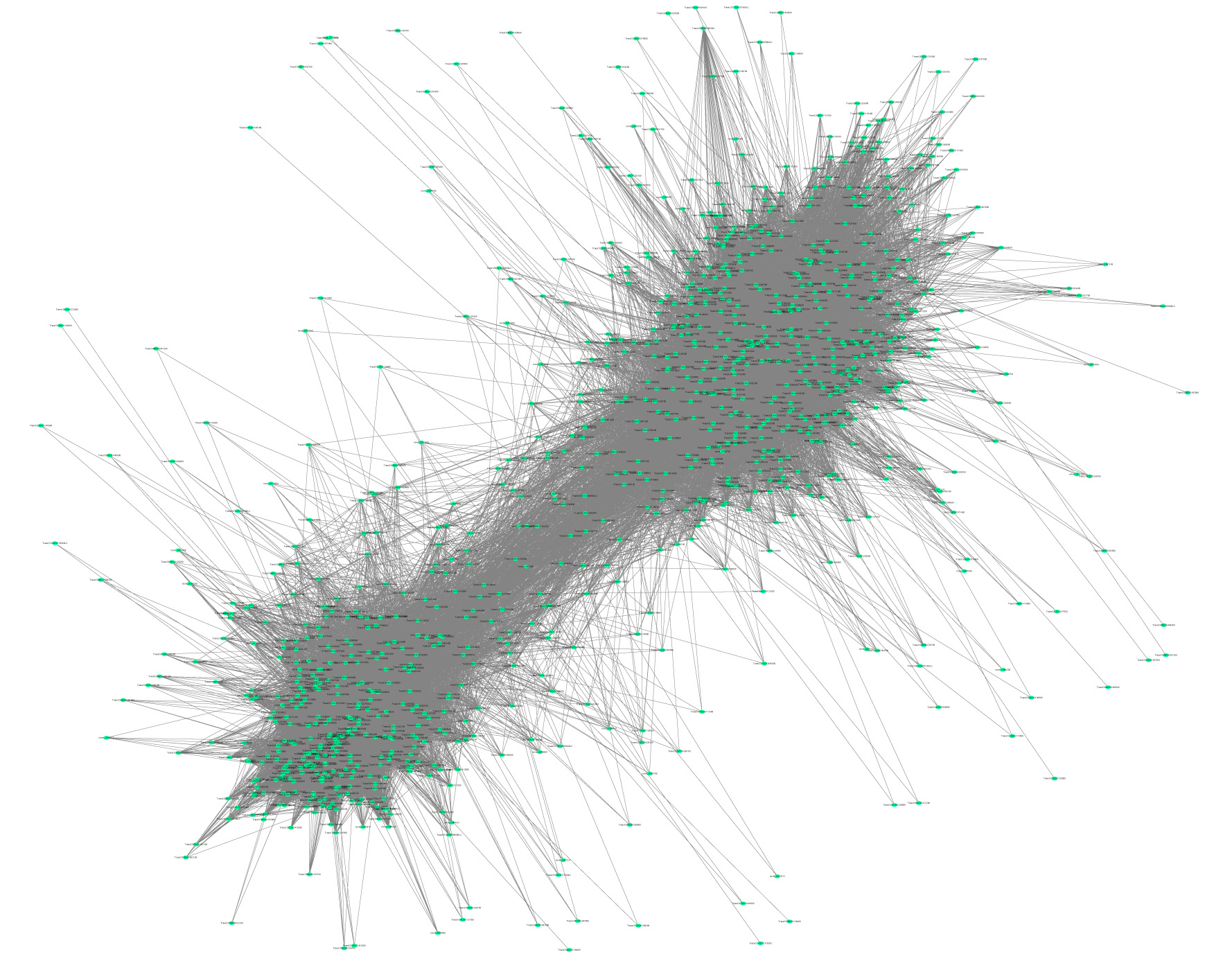


**Additional file 10: Figure S10.** Sectional area of the tapetum cell (A) and TUNEL positive nuclei/total nuclei proportion for key stages (B).

**
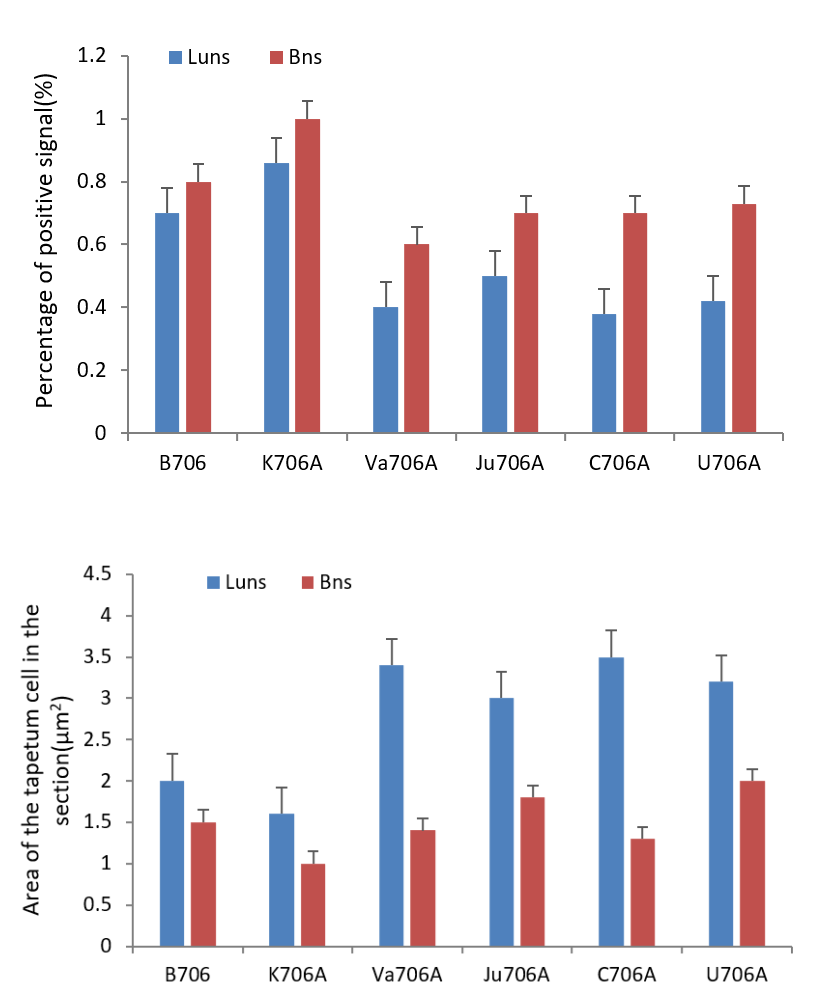
**

**Additional file 11: Figure S11.** Coefficient analysis of fold change data between qRT-PCR and RNA-seq. Data indicating relative transcript level from qRT-PCR are means of three replicates. Scatterplots were generated by log2 expression ration from RNA-seq (x-axis) and qRT-PCR (y-axis).

**
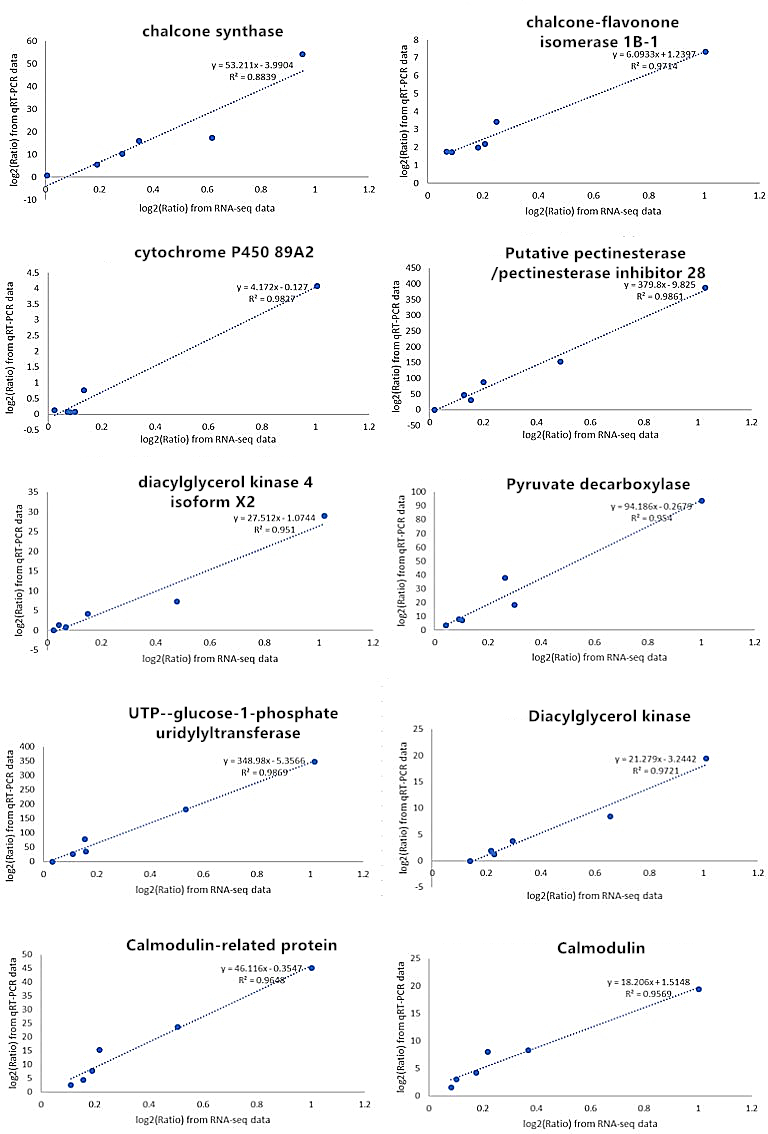
**
